# Genome-wide and Mendelian randomisation studies of liver MRI yield insights into the pathogenesis of steatohepatitis

**DOI:** 10.1016/j.jhep.2020.03.032

**Published:** 2020-08

**Authors:** Constantinos A. Parisinos, Henry R. Wilman, E. Louise Thomas, Matt Kelly, Rowan C. Nicholls, John McGonigle, Stefan Neubauer, Aroon D. Hingorani, Riyaz S. Patel, Harry Hemingway, Jimmy D. Bell, Rajarshi Banerjee, Hanieh Yaghootkar

**Affiliations:** 1 Institute of Health Informatics, Faculty of Population Health Sciences, University College London, London, UK; 2 Research Centre for Optimal Health, School of Life Sciences, University of Westminster, London, UK; 3 Perspectum Diagnostics Ltd., Oxford, UK; 4 Oxford Centre for Clinical Magnetic Resonance Research, Division of Cardiovascular Medicine, Oxford NIHR Biomedical Research Centre, University of Oxford, Oxford, UK; 5 Institute of Cardiovascular Science, Faculty of Population Health Sciences, University College London, London, UK; 6 Health Data Research UK London, Institute of Health Informatics, Faculty of Population Health Sciences, University College London, London, UK; 7 Genetics of Complex Traits, College of Medicine and Health, University of Exeter, Exeter, UK; 8 Division of Medical Sciences, Department of Health Sciences, Luleå University of Technology, Luleå, Sweden

**Keywords:** Magnetic resonance imaging, cT1, Fibrosis, Steatohepatitis, Metabolic syndrome, Genome-wide association study, Transaminases

## Abstract

**Background & Aims:**

MRI-based corrected T1 (cT1) is a non-invasive method to grade the severity of steatohepatitis and liver fibrosis. We aimed to identify genetic variants influencing liver cT1 and use genetics to understand mechanisms underlying liver fibroinflammatory disease and its link with other metabolic traits and diseases.

**Methods:**

First, we performed a genome-wide association study (GWAS) in 14,440 Europeans, with liver cT1 measures, from the UK Biobank. Second, we explored the effects of the cT1 variants on liver blood tests, and a range of metabolic traits and diseases. Third, we used Mendelian randomisation to test the causal effects of 24 predominantly metabolic traits on liver cT1 measures.

**Results:**

We identified 6 independent genetic variants associated with liver cT1 that reached the GWAS significance threshold (*p* <5×10^-8^). Four of the variants (rs759359281 in *SLC30A10*, rs13107325 in *SLC39A8*, rs58542926 in *TM6SF2*, rs738409 in *PNPLA3*) were also associated with elevated aminotransferases and had variable effects on liver fat and other metabolic traits. Insulin resistance, type 2 diabetes, non-alcoholic fatty liver and body mass index were causally associated with elevated cT1, whilst favourable adiposity (instrumented by variants associated with higher adiposity but lower risk of cardiometabolic disease and lower liver fat) was found to be protective.

**Conclusion:**

The association between 2 metal ion transporters and cT1 indicates an important new mechanism in steatohepatitis. Future studies are needed to determine whether interventions targeting the identified transporters might prevent liver disease in at-risk individuals.

**Lay summary:**

We estimated levels of liver inflammation and scarring based on magnetic resonance imaging of 14,440 UK Biobank participants. We performed a genetic study and identified variations in 6 genes associated with levels of liver inflammation and scarring. Participants with variations in 4 of these genes also had higher levels of markers of liver cell injury in blood samples, further validating their role in liver health. Two identified genes are involved in the transport of metal ions in our body. Further investigation of these variations may lead to better detection, assessment, and/or treatment of liver inflammation and scarring.

## Highlights

• Variants in metal ion transporter and NAFLD genes are associated with liver MRI-derived cT1, a steatohepatitis and fibrosis proxy.
• cT1 is highly heritable, and is correlated with BMI, NAFLD and VLDL, and inversely correlated with HDL.
• Insulin resistance, NAFLD and higher BMI are genetically linked to higher liver cT1, whilst favourable adiposity is linked to lower cT1.

## Graphical abstract

**Figure:**
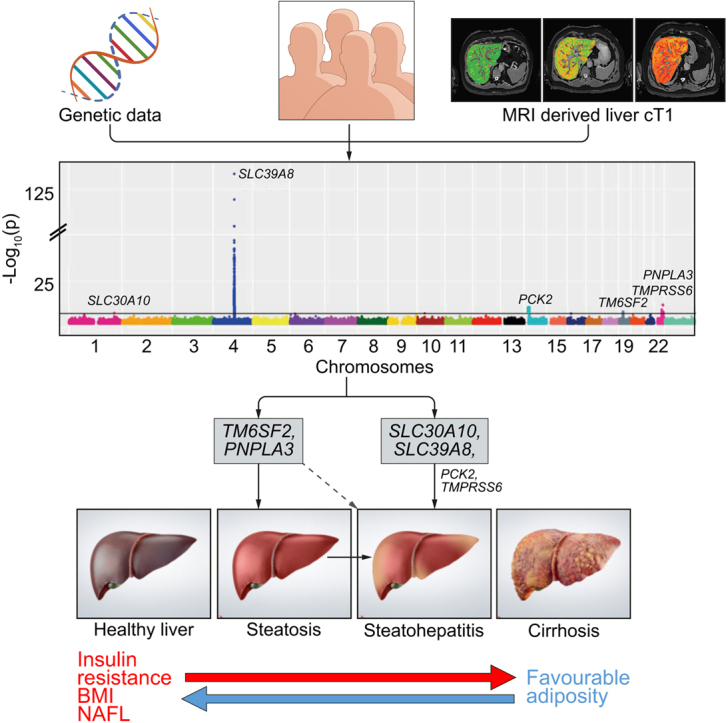

## Introduction

Non-alcoholic and alcoholic fatty liver diseases are common in an era of widespread obesity and concerning alcohol use.^1^^,^^2^ They affect up to a third of the adult population worldwide and account for the vast majority of chronic liver diseases.^3^ However, an important paradox in the history of liver fat accumulation exists; despite the large proportion of adults affected by simple steatosis (fatty liver), only a relatively small proportion (2.4–12.8%) will experience significant liver disease or liver-related death.^4^

It is important to identify which individuals are at risk of developing the more inflammatory phenotype, steatohepatitis (a condition characterised by lipotoxicity and histological necroinflammation), which is considered to be the main pathophysiological driver of liver fibrosis and subsequent disease progression.^5^ Steatohepatitis and fibrosis affect approximately 1 in 10 middle-aged adults, and can lead to cirrhosis, hepatocellular carcinoma and death.^6^

**Fig. 1:**
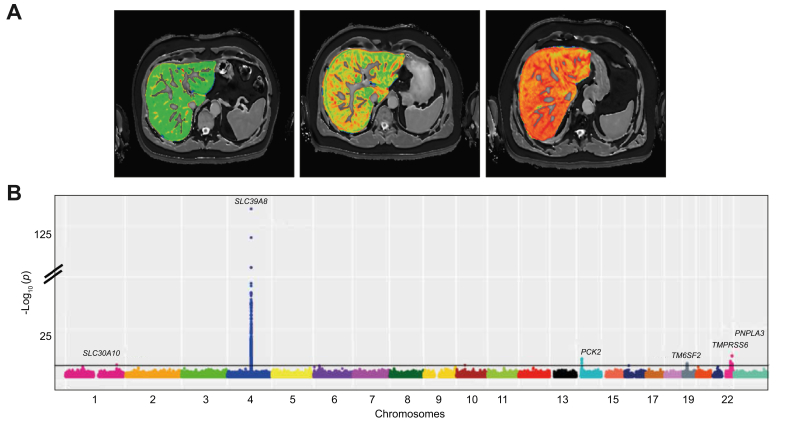
GWAS of liver cT1 in the UK Biobank. (A) Liver MRI scans of cT1. Three selected cases of liver MRI scans showing, from left to right, progressively elevated cT1 values (671 ms, 777 ms, 917 ms), reproduced by kind permission of UK Biobank©. (B) Manhattan plot illustrating GWAS of liver cT1 measurements in 14,440 UK Biobank individuals (~12 million imputed variants). The x-axis is the chromosomal position and y-axis is the significance of association for each variant in log10(*p* values). Grey line indicates genome-wide significance level. For the GWAS, a linear mixed model was used. Levels of significance: *p <*5×10^−8^_._ cT1, corrected T1; GWAS, genome-wide association study. (This figure appears in color on the web.)

A promising, non-invasive measure of steatohepatitis and fibrosis severity is MRI-based corrected T1 (cT1) (Fig. 1A).[7], [8], [9] T1 relaxation time reflects extracellular fluid, which is characteristic of fibrosis and inflammation. The presence of iron, which can be determined from T2∗ maps, has an opposing effect. Combining T2∗ and T1 values can correct for this opposing effect, from which cT1 (in milliseconds) is derived. Higher cT1 values are associated with both histological liver inflammation and fibrosis, although their relative contributions to the score are still unknown.^9^^,^^10^ cT1 has already been used as a non-invasive outcome measure in randomised controlled trials for non-alcoholic steatohepatitis (NASH)^11^ and is associated with liver disease outcomes.^8^

Understanding the underlying genetic susceptibility to steatohepatitis and fibrosis may provide new insights into the main pathophysiological mechanisms that contribute to chronic liver disease, helping in the identification of new drug targets. Genetic studies have so far been limited due to the phenotyping challenge. Liver biopsy is an invasive procedure with associated risks, significant sampling error and marked interobserver variance,^12^ while routinely available liver blood tests such as aminotransferases, despite being useful in the identification of important liver disease susceptibility loci, are overall poor predictors of liver disease severity.^13^^,^^14^

Another challenging question is which metabolic traits cause steatohepatitis since treating causal factors can help prevent liver disease. Observational associations between steatohepatitis and other features of the metabolic syndrome might occur because they share common risk factors, rather than one causing the other. Mendelian randomisation is an established epidemiological approach that uses genetic studies to provide insight on causality.^15^ Mendelian randomisation uses genetic variants associated with an exposure (*e.g.* body mass index [BMI], LDL cholesterol, insulin resistance) to assess their causal effect on an outcome of interest (*e.g.* cT1, steatohepatitis). Genetic markers of a risk factor are largely independent of confounders that may otherwise cause bias since genetic variants are randomly allocated before birth. Furthermore, the non-modifiable nature of genetic variants provides an analogy to randomised trials, in which exposure is allocated randomly and is non-modifiable by subsequent disease.^16^

In this study, we aimed to (i) identify genetic variants influencing liver cT1 (ii) identify the effect of liver cT1 variants on other metabolic traits, (iii) investigate which metabolic traits are genetically correlated with cT1 measures and (iv) use Mendelian randomisation to investigate whether 24 metabolic traits and conditions are causally associated with cT1. We performed the first genome-wide association study (GWAS) on MRI liver cT1 in 14,440 European individuals from the UK Biobank. Finally, to investigate whether there are shared variants between liver cT1 and liver fat, we carried out a GWAS on MRI determined liver proton density fat fraction (PDFF) in the same cohort.

## Materials and methods

### UK Biobank participants

UK Biobank is a prospective cohort study that consists of over 500,000 individuals aged 37–73 years (99.5% were between 40 and 69 years of age) who were recruited between 2006 and 2010 from across the UK.^17^ This research has been conducted using the data obtained via UK Biobank Access Application number 9914. The UK Biobank has approval from the North West Multi-Centre Research Ethics Committee (ref: 11/NW/0382) and obtained written informed consent from all participants prior to the study.

### Imaging protocol and analysis

Invitation to the UK Biobank imaging study is based only on proximity to one of the main imaging sites. Participants were invited and scanned at the UK Biobank Imaging Centre in Cheadle (UK) using a Siemens 1.5T Magnetom Aera as previously described.^18^^,^^19^ Medical conditions were not taken into account except from those which would exclude the participant from being able to have an MRI (*e.g.* if they had an implanted defibrillator or metal implant).

Characterisation of cT1 in the UK Biobank cohort, alongside normal values and inter- and intra-reader variability have previously been published.^18^ Briefly, 2 sequences were used to acquire data: a shortened modified look locker inversion (ShMOLLI) to quantify liver T1, and a multiecho-spoiled gradient-echo, to quantify liver iron and fat (PDFF). In both cases, data was acquired as a single transverse slice captured through the centre of the liver superior to the porta hepatis. Acquisition was performed in end-expiration breath-hold and without the aid of any contrast agent injection. The slice-based methodology has previously been shown to correlate well with histology and predict liver-related outcomes.^7^^,^^9^

The MRI sequence is part of the Liver*MultiScan*© protocol from Perspectum Diagnostics (UK) which forms part of the UK Biobank abdominal imaging protocol.^18^^,^^20^^,^^21^ The data was analysed by a team of trained analysts blinded to any participant variables, using Liver*MultiScan*© Discover 4.0 software. This software creates T2∗, cT1 and PDFF maps from the image data, and produces an automated delineation of the liver excluding its major vessels within the image slice, using a deep learning approach which has previously been published;^22^ The median value from this delineation on the T2∗ map is converted to an iron value,^23^ which is used with the ShMOLLI data to derive the cT1 map.^24^ All values reported in this work are the median, for each metric, of all usable voxels in the liver within the image slice. T1 relaxation time reflects extracellular fluid and is characteristic of fibrosis and inflammation. The presence of iron, which can be determined from T2∗ maps, has an opposing effect on the T1, and algorithms have been formed to correct for the resulting bias.^9^ All processed data are available through application to the UK Biobank. Fig. 1A illustrates the 3 MRI scans with different levels of cT1 in 3 participants.

From an initial collection of 20,386 imaging sessions (each of a unique individual), 691 did not have all the necessary imaging data, 1,354 were run with an early flawed protocol, 1,717 did not correctly trigger the sequence, 126 had more than half of their liver excluded due to poor model fitting and motion artefacts, leaving 16,498 for human quality control.

From these, a further 959 were removed through a combination of fat/water swaps, erroneous overcorrection of iron, misplacement of the image slice, segmentation failure, field artefacts, and cysts within the image slice that prevented reasonable quantification of parenchyma, leaving 15,539 participants.

### Genetic data

Protocols for the participant genotyping, data collection, and quality control have previously been described in detail.^17^ Briefly, participants were genotyped using 1 of 2 purpose-designed arrays (UK BiLEVE Axiom Array [n = 50,520] and UK Biobank Axiom Array [n = 438,692]) with 95% marker overlap. We excluded individuals who were identified by the UK Biobank as outliers based on either genotyping missingness rate or heterogeneity, or whose sex inferred from the genotypes did not match their self-reported sex. We removed individuals with a missingness >5% across variants which passed our quality control procedure. We used the latest release which included imputed data using 2 reference panels: a combined UK10K and 1000 Genomes panel and the Haplotype Reference Consortium panel. We limited our analysis to genetic variants with a minimum minor allele frequency (MAF) >1% and imputation quality score >0.3.

To define “white European” ancestry, we first used data from 1000 genomes samples to generate ancestry informative principal components (PCs). We then used these PCs in UK Biobank participants and employed K-means clustering to identify samples clustered with the 3 main 1000 genomes populations (European, African, and South Asian). Those clustered with the 1000 genomes’ “European” cluster were classified as having European ancestry.

In total, after image analysis and quality control steps, liver cT1 and PDFF measures were available for 14,440 white European individuals who also had genetic data available and were classified as white European.

### Genome-wide association analysis

We used BOLT-LMM v2.3.4 to conduct a linear mixed model GWAS which accounts for population structure and relatedness. We increased our power by including all related individuals of European descent (n = 14,440). The relatedness matrix was computed using common (MAF >5%) genotyped variants that passed quality control in all 106 batches and were present on both genotyping arrays. Prior to association testing, liver cT1 and PDFF were inverse-normal transformed. We used age, sex, centre and genotyping arrays as covariates in the model.

### Sensitivity analyses

We performed 6 sensitivity analyses (Table S1). We carried out GWASs and adjusted for (i) BMI and (ii) alcohol units consumed. We derived an alcohol units per day variable from the UK Biobank as previously suggested.^25^ In summary, a 125 ml glass of wine (red, white, or sparkling) was considered to be 1.5 units, a pint of beer or cider was considered to be 2.8 units, other alcoholic drinks (*e.g.* alcopops) were considered to be 1.5 units, and a measure of spirit was considered to be 1 unit. We further adjusted for (iii) MRI-determined liver fat and (iv) liver iron to rule out the confounding effects of these 2 traits in our image processing pipeline. Finally, we carried out GWASs in (v) males and (vi) females separately to detect sex-specific associations.

### Association of cT1 variants with liver biomarkers and metabolic traits and diseases

To further understand the role of each cT1 variant in the pathophysiology of liver disease, and also as a positive control, we tested the association between each variant and liver biomarkers in white European participants from the UK Biobank. We measured the following liver biomarkers: liver enzymes (alanine aminotransferase [ALT], aspartate aminotransferase [AST], gamma glutamyltransferase, alkaline phosphatase in up to 378,821 individuals), MRI-derived liver PDFF (n = 14,440), and MRI-derived liver iron (to understand if the correction of T1 measures for liver iron content has caused any bias; n = 14,440). The protocols for the derivation of MRI PDFF and liver iron have previously been published.^20^^,^^21^ To validate the associations with aminotransferases in a non-UK Biobank dataset, we looked up the effects of cT1 variants in an existing GWAS of ALT and AST levels in up to 61,089 individuals.^26^

To understand the effect of cT1 variants on cardiometabolic traits and diseases, we tested their associations with 15 predominantly metabolic traits including BMI, HDL-cholesterol, LDL-cholesterol, triglycerides, systolic blood pressure, diastolic blood pressure, type 2 diabetes, and coronary artery disease in up to n = 379,308 white European UK Biobank participants.

### LD score regression and cross-trait genetic correlation analysis

We used LD Hub to conduct linkage disequilibrium (LD) score regression and heritability analysis. LD Hub is a centralised database of summary level GWAS for >500 diseases and traits from publicly available resources/consortia and uses a web interface that automates LD score regression, heritability and cross-trait genetic correlation analysis.^27^ We ran heritability analysis as well as genetic correlation analysis across 120 potentially relevant traits. Single-nucleotide polymorphism (SNP)-based heritability (*h*2_SNP_) is the proportion of total variation in liver cT1 measures due to the additive genetic variation between individuals in our study population.

### Liver cirrhosis variants

To investigate the effect of liver cirrhosis variants on cT1 measures, and also as a positive control, we used variants associated with all-cause cirrhosis including rs2642438 (in or near *MARC1*), rs72613567 (*HSD17B13*), rs58542926 (*TM6SF2*), rs738409 (*PNPLA3*), rs1800562 (*HFE*), and rs28929474 (*SERPINA*).^28^

### Mendelian randomisation

We investigated the potential causal associations between 24 predominantly metabolic traits on cT1 using 2-sample Mendelian randomisation analysis.^29^ We used the inverse variance weighted approach (IVW) as our main analysis, and Mendelian randomisation-Egger and penalised weighted median as sensitivity analyses in order to detect unidentified pleiotropy of our genetic instruments. Genetic instruments were constructed by using the independent genome-wide significant genetic variants (R^2^ <0.1) of the exposure of interest from previous GWASs. For more information on Mendelian randomisation and genetic instrument selection please see the Supplementary Material.

## Results

### The characteristics of liver cT1 cohort

In our discovery cohort, median age was 57 years (interquartile range (IQR) 50–62) for males and 55 years (IQR 48–60) for females. The median liver cT1 was 694 ms (IQR 662–730) in males and 676 ms (IQR 647–710) in females (Fig. S1); 5.3% of males (299/5,595) and 2.6% of females (169/6,455) had values above 800 ms, a threshold that has been set in current clinical trials as a cut-off for steatohepatitis,^30^ and is under evaluation by the FDA and EMA as a diagnostic enrichment biomarker for NASH. Baseline characteristics were comparable to the rest of the UK Biobank cohort who did not participate in the imaging study except BMI, waist circumference and diabetes prevalence which were lower in both males and females in the liver cT1 cohort compared to the rest of the UK Biobank (Table 1). Although invitation was not based on any medical information, MRI exclusion criteria (*e.g.* metal or electrical implants, surgery 6 weeks prior to appointment, severe hearing or breathing problems) and the imaging site location (Cheadle, UK) may have contributed to a slightly healthier cohort.^21^

**Table 1:** Characteristics of UK Biobank participants in the imaging subset and the subset of participants who were not part of the imaging study.

Characteristics	UK Biobank imaging subset		UK Biobank non-imaging subset	
	Men	Women	Men	Women
n (%)	7,142	8,396	229,134	273,402
Age, years (IQR)	57 (50–62)	55 (48–60)	58 (50–64)	57 (50–63)
Waist circumference, cm (IQR)∗	94 (87–100)	79 (73–87)	96 (89–103)	83 (75–92)
Townsend deprivation index (IQR)	−2.78 (−3.98 to 0.82)	−2.66 (−3.90 to −0.69)	−2.12 (−3.65 to 0.63)	−2.14 (−3.63 to 0.49)
Self-reported diabetes (%)∗	245 (3.43%)	116 (1.38%)	15,950 (7.0%)	9,794 (3.6%)
Liver cT1, ms (IQR)	694 (662–730)	676 (647–710)	n.a.	n.a.
BMI, kg/m^2^ (IQR)∗	26.6 (24.5–28.8)	25 (22.9–28)	27.3 (25–30.1)	26.1 (23.5–30)

∗ BMI (Mann-Whitney *U* test, *p =* 1×10^−80^), waist circumference (Mann-Whitney *U* test, *p =* 1×10^−100^), diabetes prevalence (Pearson's chi squared test, *p =* 1×10^−27^) were lower in the imaging subset compared to the rest of UK Biobank. Levels of significance for all tests: (*p <*0.05).

### Genetic variants in 6 loci show association with liver cT1

In our GWAS of liver cT1 in individuals of European ancestry, variants in 6 independent loci (Table 2) reached genome-wide significance. Genomic inflation was low (*λ*_GC_ = 1.006, Fig. S2). We observed the strongest association with a missense variant, rs13107325, located in an exon of *SLC39A8* (Fig. 1B). The minor allele (T; allele frequency 7%) of rs13107325 was associated with 0.54 SD increase in cT1 (*p =* 1.2×10^−133^). The mean cT1 was 692 ms in individuals with no risk allele, 727 ms in heterozygotes, and 772 ms in risk allele homozygotes (Fig. S3).

**Table 2:** The association between 6 independent genetic variants and liver cT1. A linear mixed model was used for genetic associations (levels of significance: *p <*5×10^−8^).

SNP	CHR	Base pairs	EA	OA	EAF	Gene	Variant type	Amino acid change	BETA	Standard error	*p* value	Variance explained
**rs759359281**	1	220,100,497	C	CA	0.06	*SLC30A10*	Intron		0.137	0.026	2.8×10^-8^	0.23
**rs13107325**	4	103,188,709	T	C	0.07	*SLC39A8*	Missense	A391T	0.544	0.022	1.2×10^-133^	3.95
**rs111723834**	14	24,572,932	A	G	0.02	*PCK2, NRL*	Missense	A561G	0.291	0.046	3.0×10^-11^	0.27
**rs58542926**	19	19,379,549	T	C	0.07	*TM6SF2*	Missense, Intron	I148M	0.124	0.022	1.4×10^-8^	0.22
**rs4820268**	22	37,469,591	G	A	0.46	*TMPRSS6*	Missense	V736A	0.066	0.012	1.6×10^-9^	0.2
**rs738409**	22	44,324,727	G	C	0.21	*PNPLA3*	Missense	E167K	0.095	0.014	9.6×10^-13^	0.9

Effects are in SD.

CHR, chromosome; EA, effect allele; EAF, effect allele frequency; OA, other allele.

Other independent variants included an intronic variant (rs759359281-CA >C) in *SLC30A10* (*p =* 2.8×10^−8^), a missense variant (rs111723834-G >A) in *PCK2* (*p =* 3.0×10^−11^), a missense variant (rs4820268-A >G) in *TMPRSS6* (*p =* 1.6×10^−9^), and 2 known cirrhosis variants (rs58542926-A >G) in *TM6SF2* (*p =* 1.4×10^−8^) and (rs738409-C >G) in *PNPLA3* (*p =* 9.6×10^−13^). The 6 variants together explained 5.38% of variation in cT1 measures in white European UK Biobank participants with the *SLC39A8* variant explaining most of this variation (3.95%) (Table 2). We estimated the SNP-based heritability (*h*^2^_SNP_) of liver cT1 to be 20%. This is higher than the heritability estimated for conditions and traits such as coronary artery disease (7%),^31^ eczema (7%),^32^ body fat % (10%)^33^ and transferrin (16%), but lower than non-alcoholic fatty liver disease (NAFLD) (22–34%).^34^

We did not detect any sex-specific associations and the effects were similar between men and women (Table S1). Sensitivity analyses that further controlled for alcohol unit intake and BMI did not identify any additional signals and did not significantly change the effect size (Table S1). Sensitivity analyses that controlled for liver PDFF removed the effects of rs58542926 in *TM6SF2* and rs738409 in *PNPLA3*, suggesting that the effects of these variants on cT1 measures are mediated through liver fat accumulation (Table S1). The cT1 increasing allele (G) at *TMPRSS6*-rs4820268 is associated with lower plasma iron levels and lower liver iron.^21^ The effect of this variant on cT1 may be due to its effect on liver iron concentrations since iron has an opposing effect to T1 relaxation time. However, sensitivity analyses that controlled for liver iron only slightly attenuated its effect on cT1 (from beta = 0.066, *p =* 2×10^−9^ to beta = 0.054, *p =* 7×10^−7^) suggesting that other mechanisms are involved and that this is a true signal.

### Genetic variants in 4 loci show association with liver MRI-determined PDFF

In our GWAS of liver PDFF in 14,440 individuals of European ancestry missense variants in 4 independent loci reached genome-wide significance (rs1260326-C >T in *GCKR*, *p =* 3.9×10^−8^, rs58542926-C >T in *TM6SF2*, *p =* 6.3×10^−37^, rs429358-C >T in *APOE*, *p =* 5.6×10^−11^, rs738409-C >G in *PNPLA3*, *p =* 5.4×10^−66^ (Table S2, Fig. S4). Genomic inflation was low (*λ*_GC_ = 1.04). Two of the 4 variants (rs738409 in *PNPLA3,* rs58542926 in *TM6SF2*) were shared between PDFF and cT1 in our GWASs.

### Four of the cT1 variants are associated with higher levels of aminotransferases and demonstrate variable effects on metabolic traits and diseases

**Fig. 2:**
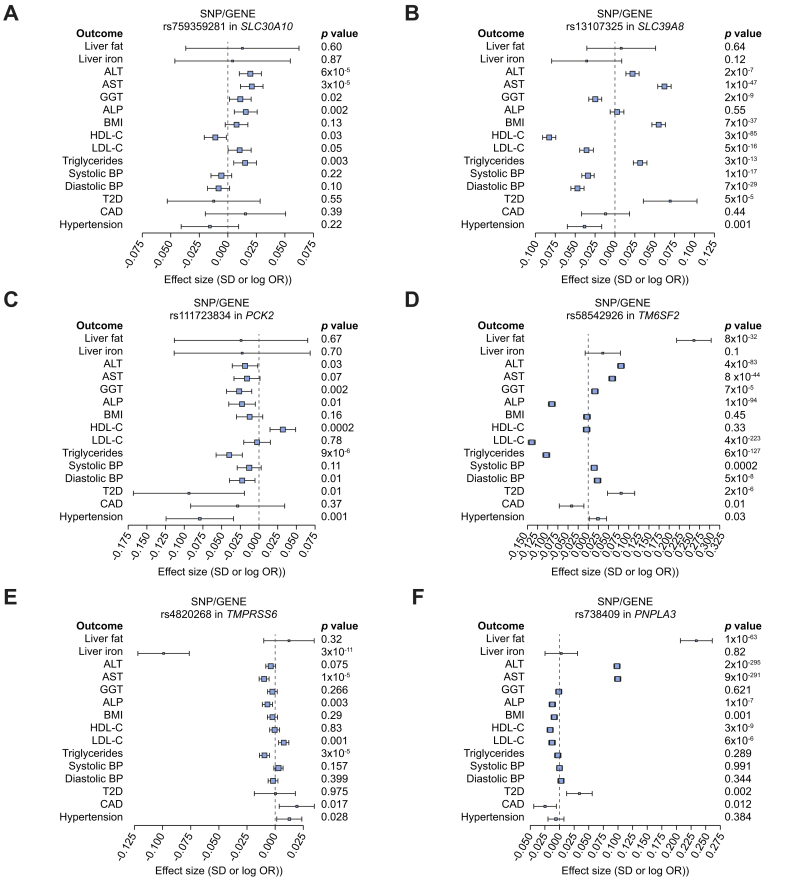
Forest plot of the associations of liver cT1 variants with liver and metabolic phenotypes. Effects are in SD for continuous traits and log(OR) for disease outcomes per copy of the risk allele. A linear mixed model was used for genetic associations. Levels of significance: *p* <0.05. ALP, alkaline phosphatase; ALT, alanine aminotransferase; AST, aspartate aminotransferase; CAD, coronary artery disease; cT1, corrected T1; GGT, gamma-glutamyltransferase; HDL-C, HDL-cholesterol; LDL-C, LDL cholesterol; OR, odds ratio; T2DM, type 2 diabetes.

To validate these variants and further understand their role in other metabolic traits and diseases, we investigated their association with liver blood tests, MRI-determined liver iron and liver PDFF, lipids, blood pressure, BMI and cardiometabolic disease outcomes (Fig. 2, Table S3). cT1-increasing alleles at 4 variants (in *SLC30A10, SLC39A8*, *TM6SF2,* and *PNPLA3*) were associated with higher ALT and AST (all with *p* values <2×10^−5^) and higher risk of type 2 diabetes (all with *p <*0.002, except the *SLC30A10* variant). None of cT1 variants were associated with cardiovascular disease risk, whilst their effects on other metabolic traits including lipids and blood pressure were variable (Fig. 2). Among the novel identified and replicated variants (rs759359281 in *SLC30A10*, and rs13107325 in *SLC39A8*), only the latter was available in a non-UK Biobank cohort with available liver blood tests. The cT1-increasing allele in rs13107325 showed a similar direction of effect on ALT (n = 46,316, beta = 0.01, *p =* 0.27) and AST (n = 39,015, beta = 0.014, *p =* 0.0005) levels in an independent cohort (Table S4).^26^

### Liver cT1 measures correlate genetically with components of metabolic syndrome

**Fig. 3:**
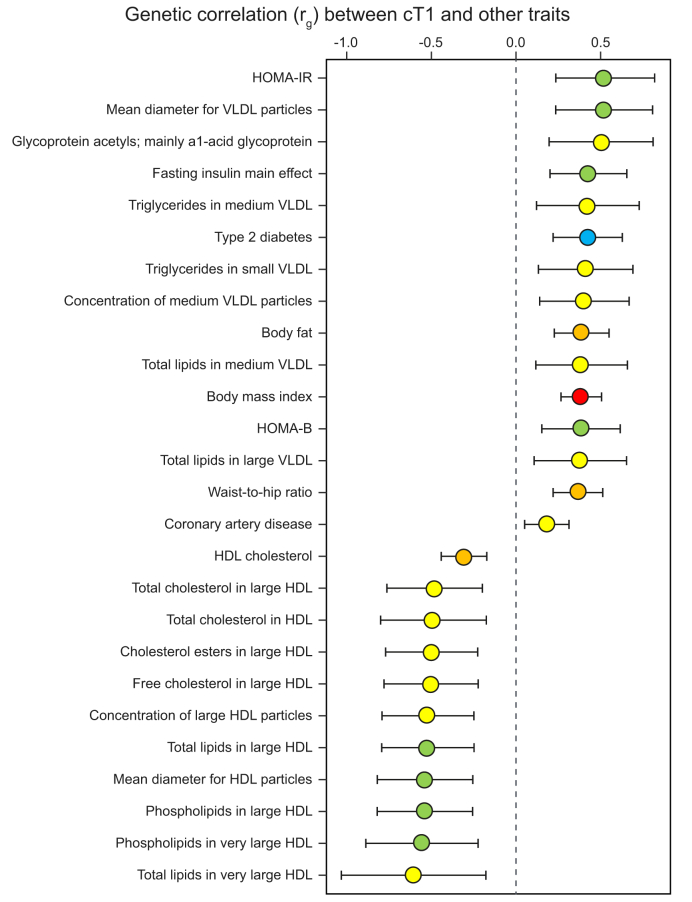
LD regression analysis. Fig. demonstrating the significant genetic correlations (rg) between cT1 and metabolic traits following correction for multiple testing (levels of significance: *p* false discovery rate <0.05) among more than 120 traits. The colours correspond to significance of correlation (*t* test); red: *p <*1×10^−8^; orange: 1×10^−6^ <*p <*1×10^−5^; blue: 1×10^−5^ <*p <*1×10^−4^; green: 1×10^−4^ <*p <*1×10^−3^; yellow: 0.001 <*p <*0.01. Higher cT1 is positively genetically correlated with VLDL, type 2 diabetes, coronary artery disease, and inversely correlated with HDL. cT1, corrected T1; HOMA-IR, homeostatic model assessment of insulin resistance, HOMA-B, homeostatic model assessment of β cell function; LD, linkage disequilibrium. (This figure appears in color on the web.)

We calculated genetic correlations using the GWAS summary statistics (120 predominantly metabolic traits/diseases) in LD score regression analysis (Fig. 3, Table S5). Measures of insulin resistance, triglycerides, VLDL, type 2 diabetes, coronary artery disease, body fat percentage, BMI and waist-to-hip ratio were positively genetically correlated with liver cT1 measures after correcting *p* values for multiple testing (false discovery rate <0.05). The most genetically correlated traits were homeostatic model for insulin resistance (HOMA-IR, *r*_*G*_ = 0.53, *p* = 0.0004) and mean diameter of VLDL particles (*r*_*G*_ = 0.52, *p* = 0.0004), whereas the strongest inverse correlation was seen with total cholesterol in very large HDL (*r*_*G*_ = -0.62, *p* = 0.04).

### Association of liver cirrhosis variants with liver cT1

We investigated the effects of all-cause cirrhosis risk variants on cT1 values. Among 6 variants associated with all-cause cirrhosis in a recent GWAS of 5,770 cases and 572,850 controls,^28^ 4 variants (those in or near *MARC1*, *HSD17B13*, *TM6SF2* and *PNPLA3*) demonstrated associations with cT1 (Table 3), where alleles associated with higher risk of liver cirrhosis were also associated with higher cT1. The *HFE* haemochromatosis risk allele (in rs1800562) was inversely associated with cT1, however this is to be expected since cT1 measures are corrected for liver iron content. Consistently, this association became remarkably attenuated (from beta = −0.11, *p =* 8×10^−7^ to beta = −0.055, *p =* 0.02) in our sensitivity analysis correcting for liver iron content. In the GWAS of all-cause cirrhosis, the effect of a1-antitrypsin risk variant (rs28929474 in *SERPINA1*) was very weak (*p =* 0.01) and present only when a recessive model was used (Table 3).^28^ We did not have any risk allele homozygotes in our liver cT1 cohort and therefore could not perform a recessive model of associations with cT1.

**Table 3:** Effects of all-cause cirrhosis risk alleles on liver cT1.

SNP	CHR	EA	OA	EAF	Beta cirrhosis†	*p* cirrhosis	Beta cT1†	SE cT1	*p* cT1	Gene
rs2642438	1	G	A	0.297	0.12	8.7×10^−7^	0.036	0.0127	0.0049	*MARC1*
rs72613567	4	T	TA	0.722	0.16	4.5×10^−8^	0.030	0.0129	0.02	*HSD17B13*
rs58542926	19	T	C	0.927	0.35	9.7×10^−24^	0.124	0.0221	1.4×10^−8^	*TM6SF2*
rs738409	22	G	C	0.211	0.38	2.2×10^−67^	0.095	0.0141	9.6×10^−13^	*PNPLA3*
rs1800562∗	6	A	G	0.925	1.16	1.3×10^−14^	-0.111	0.0223	8×10^−7^	*HFE*
rs28929474∗	14	T	C	0.0186	0.29	0.01	-0.037	0.0430	0.47	*SERPINA1*

CHR, chromosome; cT1, corrected T1; EA, effect allele; EAF, effect allele frequency; GWAS, genome-wide associated study; OA, other allele; OR, odds ratio; SNP, single-nucleotide polymorphism.

∗Indicates recessive models were run for the previously published all-cause cirrhosis GWAS; all other association analyses used additive models. Logistic regression was used for the genetic associations with cirrhosis; a linear mixed model was used for the genetic associations with cT1 (levels of significance: *p <*5x10^−8^, suggestive *p <*0.05).

† Beta cirrhosis is the effect on all-cause cirrhosis in log(OR) and Beta cT1 is the effect on cT1 in SD.

### Mendelian randomisation analysis provides genetic evidence that non-alcoholic fatty liver, insulin resistance and obesity causally elevate liver cT1

**Fig. 4:**
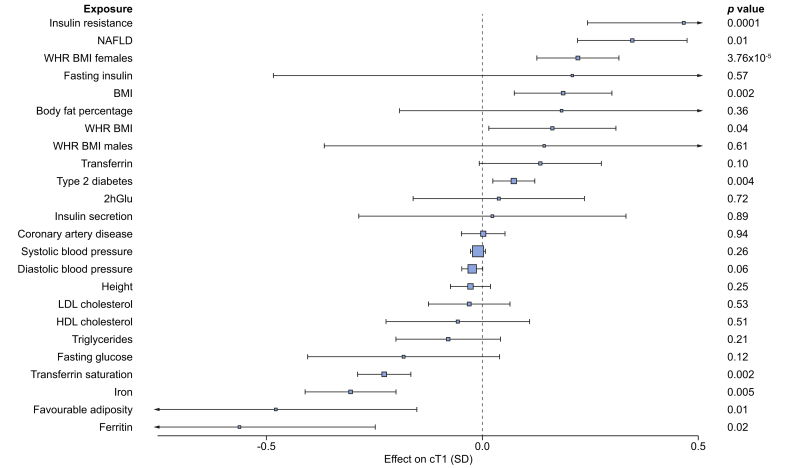
Mendelian randomisation investigating the effect of 24 predominantly metabolic traits on liver cT1. We used 2-sample Mendelian randomisation analysis to investigate the causal effects of metabolic traits on liver cT1. For full results, including sensitivity analyses, please see Table S4. The inverse variance weighted test was used as the main analysis. Levels of significance: *p <*0.05. 2hGlu, 2 hour glucose tolerance test; cT1, corrected T1; NAFLD, non-alcoholic fatty liver disease; WHR_BMI, waist hip ratio adjusted for BMI.

Demonstrating causality using observational studies can be challenging due to the presence of confounders such as other features of metabolic syndrome and behaviours including smoking and alcohol intake.^35^ In UK Biobank, we detected a strong correlation between cT1 and BMI (r^2^ = 0.36, *p =* 5×10^−324^) and also between cT1 and MRI-determined liver fat PDFF (r^2^ = 0.62, *p =* 5×10^−324^), and a weak but significant inverse correlation with liver iron (r^2^ = -0.069, *p =* 6.6×10^−18^), which is to be expected since cT1 measures were corrected for liver iron (Fig. S5). We used genetic methods (Mendelian randomisation, Fig. 4) that are generally free of biases such as confounding and reverse causation to examine the potential causal effect of metabolic traits on liver cT1. We found evidence of a causal association between insulin resistance (IVW *p =* 0.0001), non-alcoholic fatty liver (IVW *p* = 0.01), type 2 diabetes (IVW *p =* 0.004), BMI (IVW *p =* 0.002) and higher cT1. We also found evidence for a protective role of favourable adiposity variants (variants associated with higher adiposity but lower risk of cardiometabolic diseases and lower ectopic fat)^36^ and cT1 (IVW *p =* 0.01) (Table S6). Our analyses were robust across a range of sensitivity analyses (Table S6).

## Discussion

We identified associations between 6 independent genetic variants and MRI-based liver cT1, a non-invasive marker of liver inflammation and fibrosis, in 14,440 participants from the UK Biobank. These include 5 missense variants (in *SLC39A8*, *PCK2*, *TM6SF2*, *PNPLA3*, and *TMPRSS6*) and 1 intronic variant (in *SLC30A10*). The cT1-increasing alleles in 4 genes (*SLC39A8, SLC30A10, PNPLA3,* and *TM6SF2*) were also associated with higher AST (n = 360,731) and higher ALT (n = 361,940) in the UK Biobank and also in an independent GWAS of liver enzymes (except for *SLC30A10* where data was not available).^26^ *SLC30A10* and *SLC39A8* encode metal ion transporters and *PNPLA3* and *TM6SF2* are known genes associated with fatty liver and cirrhosis.

cT1 is a continuous trait, and was analysed as such in our GWAS, in line with other continuous traits such as blood pressure, BMI and height.[37], [38], [39] In some earlier publications, cT1 was reported using the LIF (liver inflammation and fibrosis) score (Supplementary Material). The LIF score is a tri-linear mapping of cT1 onto a continuous scale from 0 to 4 based on the association of cT1 with histological fibrosis.^9^ LIF categories were defined as having no (LIF <1), mild (LIF 1–1.99), moderate (LIF 2–2.99), or severe (LIF 3–4) liver disease.^8^ The LIF cut-off of 1.4 had a sensitivity of 91% and a specificity of 52% for the diagnosis of NASH *vs.* steatosis (AUROC = 0.80), and corresponds to a cT1 value of 780 ms; a slightly higher cut-off of 800 ms is used in clinical trials^30^ and is under evaluation by the FDA and EMA as a diagnostic enrichment biomarker for NASH.^9^^,^^40^ The LIF score is no longer used since the medical and MRI physics community is more familiar with T1 for the assessment of inflammation and fibrosis across all specialties including cardiology and neurology.^11^^,^^18^^,^[41], [42], [43], [44], [45] In this GWAS study, the cT1 values reported are standardised across the MRI scanner model and field strength, showing very high repeatability and reproducibility.^46^

The missense variant (rs13107325-C >T) in *SLC39A8* is predicted to be deleterious in both Polyphen-2 and SIFT, and is associated with lower expression of *SLC39A8* in human liver.^47^ *SLC39A8* encodes ZIP8, which has important roles in inflammation and immunity, and is a negative regulator of the NF-kB pathway.^48^ ZIP8 is a divalent cation importer capable of transporting zinc, manganese, iron, cadmium and selinate; the substitution of C for T allele impairs the cellular uptake of metals by this protein.^49^ It is not known which metal is involved in liver pathogenicity but there is evidence that hepatic ZIP8 regulates manganese metabolism in the liver, a metal ion that is hepatotoxic at high levels.^50^ Zinc and selenium also have important roles in liver cellular injury, oxidative stress and dysregulated inflammation; dietary supplementation of both has shown benefit in animal models of liver disease.^51^^,^^52^

The pathogenic role of *SLC39A8* in liver inflammation and fibrosis is supported by studies in mice which provide mechanistic evidence for the critical role of ZIP8 in liver disease. Liu *et al.*^53^ used 2 mouse models to study the function of *SLC39A8* in the liver. In the first model, they studied the chronic effect of *SLC39A8* knockdown. The *SLC39A8(neo/neo)* homozygous mice died before or immediately after birth. The *SLC39A8(+/neo)* heterozygous mice had moderate ZIP8 deficiency which led to disruption of normal hepatocellular architecture, necrosis, inflammation, fibrosis and development of liver tumours with histopathological features consistent with hepatocellular neoplasms.^53^ In the second model, they studied liver-specific *SLC39A8* knockdown by adenovirus-delivered short-hairpin RNA and demonstrated that liver damage in the chronic model is not due to some extrahepatic process. Liver-specific ZIP8 downregulation for 7 days resulted in substantial hepatomegaly, inflammation, proliferation, oxidative stress, liver injury and cell death.^53^

The intronic variant in *SLC30A10,* a gene which codes a predominantly manganese metal ion transporter, was also associated with elevated cT1 measures in our study, as well as elevated aminotransferases in the UK Biobank. Manganese is an essential metal required for the adequate functioning of numerous enzymes, however it is toxic and induces cell death at elevated cellular levels.^54^ Loss-of-function mutations in *SLC30A10* have previously been associated with cirrhosis, higher manganese levels in liver biopsy samples and neurotoxicity including parkinsonian-like movement disorders.^54^^,^^55^

The association between cT1 increasing alleles at the 2 novel loci (*SLC39A8* and *SLC30A10)* and higher ALT and AST adds supportive evidence for their pathogenic role in the liver. The missense variant in *SLC39A8* has previously been shown to be associated with multiple traits including alcohol intake, BMI, schizophrenia, Crohn's disease, lower brain grey matter volume and microbiome diversity;^38^^,^[56], [57], [58] we show for the first time a further novel association with higher diabetes and triglyceride levels, whilst highlighting variable effects on cholesterol levels. The associations of both variants with cT1 were independent of BMI, alcohol intake, liver fat percentage and liver iron content in our sensitivity models.

We identified a further 2 missense variants that were associated with cT1 but not with elevated aminotransferases; therefore, further research is required to validate these findings and explore their potential role in liver inflammation and fibrosis. The cT1-increasing allele in rs111723834 (missense variant in *PCK2,* also an intronic variant in *NRL*) was associated with lower aminotransferases, lower risk of type 2 diabetes, and lower triglycerides. *PCK2* encodes a mitochondrial enzyme that catalyses the conversion of oxaloacetate to phosphoenolpyruvate and has a key role in hepatic gluconeogenesis. Mitochondrial phosphoenolpyruvate carboxykinase deficiency (M-PEPCKD) is a rare autosomal recessive disorder resulting from impaired gluconeogenesis, and clinical characteristics include hypotonia, hepatomegaly, failure to thrive, lactic acidosis and hypoglycaemia.^59^ The missense variant in *PCK2* is also an intronic variant in *NRL,* and it is unclear which gene is associated with elevated cT1 measures. *NRL* however encodes for neural retinal leucine zipper transcription factor that is specifically expressed in neuronal retina cells, making it an unlikely causal gene candidate for liver cT1. The cT1-increasing allele (rs4820268-A >G) in *TMPRSS6* has previously been reported to be associated with lower plasma iron levels and lower liver iron content.^21^^,^^60^ It is also associated with a dysmetabolic profile including higher LDL cholesterol, higher cardiovascular disease risk and hypertension (Fig. 2). Its effect on cT1 however remained significant even after correcting for liver iron content in sensitivity analyses, making it unlikely that the association was secondary to bias resulting from iron correction when calculating cT1. Previous Mendelian randomisation studies have shown that higher circulating iron may be cardioprotective,^61^ possibly through reduced circulating LDL-cholesterol and lower blood pressure.^62^ The same mechanisms may explain why the allele associated with lower circulating iron levels is associated with higher cT1.

Known NAFLD and cirrhosis risk alleles in *PNPLA3* and *TM6SF2* were also associated with both elevated cT1 and MRI-derived PDFF in our cohort. These associations provide strong positive controls for our study and validate for the first time the association with MRI-determined liver PDFF. The risk alleles in these 2 genes were further associated with higher risk of type 2 diabetes, but with lower serum triglycerides, LDL cholesterol, and lower risk for cardiovascular disease, as previously described.^63^^,^^64^ In our GWAS on PDFF, alongside *PNPLA3* and *TM6SF2*, we further identified variants in *GCKR* (another known NAFLD variant which we have replicated) and *APOE* (apolipoprotein E, a gene which encodes a major cholesterol carrier).^63^^,^^65^ The *APOE* risk allele (T) for PDFF is associated with a higher risk of diabetes, and lower risk of cardiovascular disease and elevated LDL cholesterol in independent GWASs.^66^ This data provide evidence that cT1 and PDFF phenotypes share some but not all aetiopathogenic mechanisms.

We demonstrated that 4 of 5 variants associated with all-cause liver cirrhosis (in *PNPLA3*, *TM6SF2, HSD17B13*, and *MARC1*)^28^ were also associated with liver cT1 with the first 2 reaching genome-wide significance. The paradoxical inverse association between the liver iron-increasing allele in *HFE* may be due to overcorrection since cT1 measures are corrected for liver iron content and were inversely correlated in our cohort. Adjustment for liver iron content in our sensitivity analysis remarkably attenuated the association with cT1. The *SERPINA1* variant was only associated with all-cause cirrhosis in a recessive model.^28^ We did not have any homozygotes in our liver cT1 cohort to detect a recessive model of association with cT1.

Identifying causal mechanisms to steatohepatitis is crucial since interventions targeting these modifiable exposures may prevent liver disease progression. Our Mendelian randomisation study investigated 24 possible metabolic traits that may cause steatohepatitis. We provide genetic evidence that insulin resistance, non-alcoholic fatty liver and higher BMI causally increase cT1. Recent genetic studies have further identified variants associated with higher BMI but lower risk of type 2 diabetes, hypertension and heart disease.^67^ These “favourable adiposity” variants are also associated with higher subcutaneous-to-visceral adipose tissue ratio and may protect from disease through higher adipose storage capacity, by promoting lipid deposition in subcutaneous tissue rather than within the circulation and ectopic places. The inverse link between favourable adiposity and steatohepatitis provides supportive evidence for the protective effects of this phenotype on a variety of cardiometabolic diseases, underlying mechanisms which can be further explored and pointing to future preventive and therapeutic strategies.

Our study had several limitations. We did not have any independent cohort to replicate our findings. To overcome this limitation, we investigated associations between cT1 variants and ALT and AST levels both in the UK Biobank and an independent GWAS of liver enzymes.^68^ While MRI-derived cT1 is clinically available and is used to assess the severity of steatohepatitis, this measure is still novel, and further research is needed to determine the relative contributions of inflammation and fibrosis to cT1.^10^ Whilst it would be useful to have histological reference data for cT1, pathologist-interpreted liver biopsies do not lend themselves to large studies of this nature because of the risk to patients and inter-rater variance in assessment of histology. This may be improved with advances in digitally processed histology and centralised collection of pathological data for large consortia like the European LITMUS study. While cT1 has demonstrated excellent repeatability^42^^,^^46^ and good correlation with fibro-inflammation and clinical outcomes,^7^^,^^9^ other histological phenomena such as simple steatosis and ballooning have been shown to contribute to an increased T1 signal.^7^ Only 2 of the 6 cT1 variants were associated with liver steatosis, which highlights the complementarity of cT1 and liver fat PDFF as biomarkers of liver status, and their potential to recognise different mechanisms predisposing individuals to liver disease.

### Conclusion

cT1 and PDFF phenotypes share some but not all aetiopathogenic mechanisms. We identified novel associations between an MRI-derived measure of fibroinflammatory liver disease and variants in *SLC30A10* and *SLC39A8* that replicated with blood biomarkers of hepatocyte injury. These genes have a critical role in transporting heavy metal cofactors for a multitude of biological processes. Future studies may determine whether targeting *SLC30A10* and *SLC39A8* are possible therapeutic options to prevent liver disease in at-risk individuals. Our Mendelian randomisation study provides genetic evidence that addressing weight gain and insulin resistance are useful strategies in the prevention of steatohepatitis.

### Abbreviations

ALP, alkaline phosphatase; ALT, alanine aminotransferase; AST, aspartate aminotransferase; BMI, body mass index; CAD, coronary artery disease; cT1, corrected T1; EA, effect allele; EAF, effect allele frequency; GGT, gamma-glutamyltransferase; GWAS, genome-wide association study; HDL-C, HDL-cholesterol; IVW, inverse variance weighted; LD, linkage disequilibrium; LDL-C, LDL cholesterol; LIF, liver inflammation and fibrosis; NAFLD, non-alcoholic fatty liver disease; NASH, non-alcoholic steatohepatitis; PC, principal components; PDFF, proton density fat fraction; OA, other allele; OR, odds ratio; ShMOLLI, shortened modified look locker inversion; SNP, single-nucleotide polymorphism; T2DM, type 2 diabetes; WHR_BMI, waist hip ratio adjusted for BMI.

## Financial support

H.Y. is funded by a Diabetes UK RD Lawrence fellowship (17/0005594). C.A.P is funded by a 10.13039/100010269Wellcome Trust Clinical PhD Programme (206274/Z/17/Z). H.W is funded by an Innovate UK Knowledge Transfer Partnership (KTP10271). H.H is a National Institute for Health Research Senior Investigator.

## Authors' contributions

C.A.P, H.W, H.Y, R.B. were involved in the study conception and design, analysis and writing of the manuscript. J.M and R.C.N were involved in analysis. H.H, A.D.H, R.S.P, C.A.P. E.L.T, J.B. edited the manuscript. S.N, R.B, M.K. edited the manuscript and provided the infrastructure underlying the MRI liver cT1 measurements.

## Ethical approval

This research has been conducted using data from the UK Biobank resource and carried out under UK Biobank project application number 9914. UK Biobank protocols were approved by the National Research Ethics Service Committee.

## Patient consent

No participants were directly involved in our study, as we used data derived from the UK Biobank study, under project application number 9914. For the UK Biobank overall study, participants signed written informed consent, specifically applicable to health-related research. All ethical regulations were followed. No patients or participants were specifically or directly involved in setting the research question or the outcome measures or in developing plans for recruitment, design, or implementation of this study. No patients were asked to advise on interpretation or drafting of results. There are no specific plans to disseminate the research results to study participants, but the UK Biobank disseminates key findings from projects on its website.

## Data availability

Full data including individual cT1 and PDFF measures will be returned to UK Biobank and made publicly available via application (amsportal.ukbiobank.ac.uk).

## Conflict of interest

M.K, J.M, R.C.N and R.B. are employees and shareholders of Perspectum Diagnostics. H.W. and S.N. are shareholders in Perspectum Diagnostics.

## Supplementary data

Supplementary information.pdf

CTAT table.pdf

## References

[1] Gilmore I., Williams R. (2019). Alcohol policy in the UK: where next?. Lancet.

[2] Younossi Z., Tacke F., Arrese M., Sharma B.C., Mostafa I., Bugianesi E. (2018). Global perspectives on non-alcoholic fatty liver disease and non-alcoholic steatohepatitis. Hepatology.

[3] Harris R., Harman D.J., Card T.R., Aithal G.P., Guha I.N. (2017). Prevalence of clinically significant liver disease within the general population, as defined by non-invasive markers of liver fibrosis: a systematic review. Lancet Gastroenterol Hepatol.

[4] White D.L., Kanwal F., El-Serag H.B. (2012). Association between nonalcoholic fatty liver disease and risk for hepatocellular cancer, based on systematic review. Clin Gastroenterol Hepatol.

[5] Day C.P., Saksena S. (2002). Non-alcoholic steatohepatitis: definitions and pathogenesis. J Gastroenterol Hepatol.

[6] Williams C.D., Stengel J., Asike M.I., Torres D.M., Shaw J., Contreras M. (2011). Prevalence of nonalcoholic fatty liver disease and nonalcoholic steatohepatitis among a largely middle-aged population utilizing ultrasound and liver biopsy: a prospective study. Gastroenterology.

[7] Pavlides M., Banerjee R., Tunnicliffe E.M., Kelly C., Collier J., Wang L.M. (2017). Multiparametric magnetic resonance imaging for the assessment of non-alcoholic fatty liver disease severity. Liver Int.

[8] Pavlides M., Banerjee R., Sellwood J., Kelly C.J., Robson M.D., Booth J.C. (2016). Multiparametric magnetic resonance imaging predicts clinical outcomes in patients with chronic liver disease. J Hepatol.

[9] Banerjee R., Pavlides M., Tunnicliffe E.M., Piechnik S.K., Sarania N., Philips R. (2014). Multiparametric magnetic resonance for the non-invasive diagnosis of liver disease. J Hepatol.

[10] Cheung A., Neuschwander-Tetri B.A., Kleiner D.E., Schabel E., Rinella M., Harrison S. (2019). Defining improvement in nonalcoholic steatohepatitis for treatment trial endpoints: recommendations from the liver forum. Hepatology.

[11] Harrison S.A., Rossi S.J., Paredes A.H., Trotter J.F., Bashir M.R., Guy C.D. (2019). NGM282 improves liver fibrosis and histology in 12 weeks in patients with nonalcoholic steatohepatitis. Hepatology.

[12] Pavlides M., Birks J., Fryer E., Delaney D., Sarania N., Banerjee R. (2017). Interobserver variability in histologic evaluation of liver fibrosis using categorical and quantitative scores. Am J Clin Pathol.

[13] Chalasani N., Younossi Z., Lavine J.E., Diehl A.M., Brunt E.M., Cusi K. (2012). The diagnosis and management of non-alcoholic fatty liver disease: practice guideline by the American Gastroenterological Association, American Association for the Study of Liver Diseases, and American College of Gastroenterology. Gastroenterology.

[14] Mofrad P., Contos M.J., Haque M., Sargeant C., Fisher R.A., Luketic V.A. (2003). Clinical and histologic spectrum of nonalcoholic fatty liver disease associated with normal ALT values. Hepatology.

[15] Davies N.M., Holmes M.V., Davey Smith G. (2018). Reading Mendelian randomisation studies: a guide, glossary, and checklist for clinicians. BMJ.

[16] Hingorani A., Humphries S. (2005). Nature's randomised trials. Lancet.

[17] Sudlow C., Gallacher J., Allen N., Beral V., Burton P., Danesh J. (2015). UK Biobank: an open access resource for identifying the causes of a wide range of complex diseases of middle and old age. PLoS Med.

[18] Mojtahed A., Kelly C.J., Herlihy A.H., Kin S., Wilman H.R., McKay A. (2019). Reference range of liver corrected T1 values in a population at low risk for fatty liver disease-a UK Biobank sub-study, with an appendix of interesting cases. Abdom Radiol (NY).

[19] Petersen S.E., Matthews P.M., Bamberg F., Bluemke D.A., Francis J.M., Friedrich M.G. (2013). Imaging in population science: cardiovascular magnetic resonance in 100,000 participants of UK Biobank - rationale, challenges and approaches. J Cardiovasc Magn Reson.

[20] Wilman H.R., Kelly M., Garratt S., Matthews P.M., Milanesi M., Herlihy A. (2017). Characterisation of liver fat in the UK Biobank cohort. PLoS One.

[21] Wilman H.R., Parisinos C.A., Atabaki-Pasdar N., Kelly M., Louise Thomas E., Neubauer S. (2019). Genetic studies of abdominal MRI data identify genes regulating hepcidin as major determinants of liver iron concentration. J Hepatol.

[22] Irving B., Hutton C., Dennis A., Vikal S., Mavar M., Kelly M. (2017). Deep quantitative liver segmentation and vessel exclusion to assist in liver assessment. Medical Image Understanding and Analysis.

[23] Wood J.C., Enriquez C., Ghugre N., Tyzka J.M., Carson S., Nelson M.D. (2005). MRI R2 and R2∗ mapping accurately estimates hepatic iron concentration in transfusion-dependent thalassemia and sickle cell disease patients. Blood.

[24] Mojtahed A., Gee M.S. (2018). Magnetic resonance enterography evaluation of Crohn disease activity and mucosal healing in young patients. Pediatr Radiol.

[25] Frayling T.M., Beaumont R.N., Jones S.E., Yaghootkar H., Tuke M.A., Ruth K.S. (2018). A common allele in FGF21 associated with sugar intake is associated with body shape, lower total body-fat percentage, and higher blood pressure. Cell Rep.

[26] Chambers J.C., Zhang W., Sehmi J., Li X., Wass M.N., Van der Harst P. (2011). Genome-wide association study identifies loci influencing concentrations of liver enzymes in plasma. Nat Genet.

[27] Zheng J., Erzurumluoglu A.M., Elsworth B.L., Kemp J.P., Howe L., Haycock P.C. (2017). LD Hub: a centralized database and web interface to perform LD score regression that maximizes the potential of summary level GWAS data for SNP heritability and genetic correlation analysis. Bioinformatics.

[28] Emdin C.A., Haas M., Khera A.V., Aragam K., Chaffin M., Jiang L. (2020). A missense variant in Mitochondrial Amidoxime Reducing Component 1 gene and protection against liver disease. PLoS Genetics.

[29] Pierce B.L., Burgess S. (2013). Efficient design for Mendelian randomization studies: subsample and 2-sample instrumental variable estimators. Am J Epidemiol.

[30] (2019). Non-invasive Rapid Assessment of NAFLD Using Magnetic Resonance Imaging With LiverMultiScan - Full Text View - ClinicalTrials.gov. https://clinicaltrials.gov/ct2/show/NCT03289897.

[31] Nikpay M., Goel A., Won H.-H., Hall L.M., Willenborg C., Kanoni S. (2015). A comprehensive 1,000 genomes-based genome-wide association meta-analysis of coronary artery disease. Nat Genet.

[32] Paternoster L., Standl M., Waage J., Baurecht H., Hotze M., Strachan D.P. (2015). Multi-ancestry genome-wide association study of 21,000 cases and 95,000 controls identifies new risk loci for atopic dermatitis. Nat Genet.

[33] Lu Y., Day F.R., Gustafsson S., Buchkovich M.L., Na J., Bataille V. (2016). New loci for body fat percentage reveal link between adiposity and cardiometabolic disease risk. Nat Commun.

[34] Palmer N.D., Musani S.K., Yerges-Armstrong L.M., Feitosa M.F., Bielak L.F., Hernaez R. (2013). Characterization of European ancestry nonalcoholic fatty liver disease-associated variants in individuals of African and Hispanic descent. Hepatology.

[35] Younossi Z.M. (2018). Non-alcoholic fatty liver disease-a global public health perspective. J Hepatol.

[36] Ji Y., Yiorkas A.M., Frau F., Mook-Kanamori D., Staiger H., Thomas E.L. (2019). Genome-wide and abdominal MRI data provide evidence that a genetically determined favorable adiposity phenotype is characterized by lower ectopic liver fat and lower risk of type 2 diabetes, heart disease, and hypertension. Diabetes.

[37] Giri A., Hellwege J.N., Keaton J.M., Park J., Qiu C., Warren H.R. (2019). Trans-ethnic association study of blood pressure determinants in over 750,000 individuals. Nat Genet.

[38] Yengo L., Sidorenko J., Kemper K.E., Zheng Z., Wood A.R., Weedon M.N. (2018). Meta-analysis of genome-wide association studies for height and body mass index in ~700000 individuals of European ancestry. Hum Mol Genet.

[39] Cantor R.M., Lange K., Sinsheimer J.S. (2010). Prioritizing GWAS results: a review of statistical methods and recommendations for their application. Am J Hum Genet.

[40] Pavlides M., Banerjee R., Kelly C.J., Robson M.D., Neubauer S., Barnes E. (2017). Reply to: “Multiparametric magnetic resonance imaging to predict clinical outcomes in patients with chronic liver disease: a cautionary note on a promising technique”. J Hepatol.

[41] Harrison S.A., Dennis A., Fiore M.M., Kelly M.D., Kelly C.J., Paredes A.H. (2018). Utility and variability of three non-invasive liver fibrosis imaging modalities to evaluate efficacy of GR-MD-02 in subjects with NASH and bridging fibrosis during a phase-2 randomized clinical trial. PLoS One.

[42] McDonald N., Eddowes P.J., Hodson J., Semple S.I.K., Davies N.P., Kelly C.J. (2018). Multiparametric magnetic resonance imaging for quantitation of liver disease: a two-centre cross-sectional observational study. Sci Rep.

[43] Ostovaneh M.R., Ambale-Venkatesh B., Fuji T., Bakhshi H., Shah R., Murthy V.L. (2018). Association of liver fibrosis with cardiovascular diseases in the general population: the Multi-Ethnic Study of Atherosclerosis (MESA). Circ Cardiovasc Imaging.

[44] Liu C.-Y., Liu Y.-C., Wu C., Armstrong A., Volpe G.J., van der Geest R.J. (2013). Evaluation of age-related interstitial myocardial fibrosis with cardiac magnetic resonance contrast-enhanced T1 mapping: MESA (Multi-Ethnic Study of Atherosclerosis). J Am Coll Cardiol.

[45] Mealer R.G., Jenkins B.G., Chen C.-Y., Daly M.J., Ge T., Lehoux S. (2019). A schizophrenia risk locus alters brain metal transport and plasma glycosylation. bioRxiv.

[46] Bachtiar V., Kelly M.D., Wilman H.R., Jacobs J., Newbould R., Kelly C.J. (2019). Repeatability and reproducibility of multiparametric magnetic resonance imaging of the liver. PLoS One.

[47] Speliotes E.K., Willer C.J., Berndt S.I., Monda K.L., Thorleifsson G., Jackson A.U. (2010). Association analyses of 249,796 individuals reveal 18 new loci associated with body mass index. Nat Genet.

[48] Liu M.-J., Bao S., Gálvez-Peralta M., Pyle C.J., Rudawsky A.C., Pavlovicz R.E. (2013). ZIP8 regulates host defense through zinc-mediated inhibition of NF-κB. Cell Rep.

[49] Haller G., McCall K., Jenkitkasemwong S., Sadler B., Antunes L., Nikolov M. (2018). A missense variant in SLC39A8 is associated with severe idiopathic scoliosis. Nat Commun.

[50] Lin W., Vann D.R., Doulias P.-T., Wang T., Landesberg G., Li X. (2017). Hepatic metal ion transporter ZIP8 regulates manganese homeostasis and manganese-dependent enzyme activity. J Clin Invest.

[51] Mousavi S.N., Faghihi A., Motaghinejad M., Shiasi M., Imanparast F., Amiri H.L. (2018). Zinc and Selenium Co-supplementation reduces some lipid peroxidation and angiogenesis markers in a rat model of NAFLD-fed high fat diet. Biol Trace Elem Res.

[52] Kang X., Zhong W., Liu J., Song Z., McClain C.J., Kang Y.J. (2009). Zinc supplementation reverses alcohol-induced steatosis in mice through reactivating hepatocyte nuclear factor-4alpha and peroxisome proliferator-activated receptor-alpha. Hepatology.

[53] Liu L., Geng X., Cai Y., Copple B., Yoshinaga M., Shen J. (2018). Hepatic ZIP8 deficiency is associated with disrupted selenium homeostasis, liver pathology, and tumor formation. Am J Physiol Gastrointest Liver Physiol.

[54] Mukhopadhyay S. (2018). Familial manganese-induced neurotoxicity due to mutations in SLC30A10 or SLC39A14. Neurotoxicology.

[55] Tuschl K., Clayton P.T., Gospe S.M., Gulab S., Ibrahim S., Singhi P. (2012). Syndrome of hepatic cirrhosis, dystonia, polycythemia, and hypermanganesemia caused by mutations in SLC30A10, a manganese transporter in man. Am J Hum Genet.

[56] Li D., Achkar J.-P., Haritunians T., Jacobs J.P., Hui K.Y., D'Amato M. (2016). A pleiotropic missense variant in SLC39A8 is associated with Crohn's disease and human gut microbiome composition. Gastroenterology.

[57] Costas J. (2018). The highly pleiotropic gene SLC39A8 as an opportunity to gain insight into the molecular pathogenesis of schizophrenia. Am J Med Genet B Neuropsychiatr Genet.

[58] Elliott L.T., Sharp K., Alfaro-Almagro F., Shi S., Miller K.L., Douaud G. (2018). Genome-wide association studies of brain imaging phenotypes in UK Biobank. Nature.

[59] Hommes F.A., Bendien K., Elema J.D., Bremer H.J., Lombeck I. (1976). Two cases of phosphoenolpyruvate carboxykinase deficiency. Acta Paediatr Scand.

[60] Benyamin B., Esko T., Ried J.S., Radhakrishnan A., Vermeulen S.H., Traglia M. (2014). Novel loci affecting iron homeostasis and their effects in individuals at risk for hemochromatosis. Nat Commun.

[61] Gill D., Del Greco M.F., Walker A.P., Srai S.K.S., Laffan M.A., Minelli C. (2017). The effect of iron status on risk of coronary artery disease: a Mendelian randomization study-brief report. Arterioscler Thromb Vasc Biol.

[62] Gill D., Brewer C.F., Monori G., Trégouët D.-A., Franceschini N., Giambartolomei C. (2019). Effects of genetically determined iron status on risk of venous thromboembolism and carotid atherosclerotic disease: a Mendelian randomization study. J Am Heart Assoc.

[63] Kahali B., Liu Y.-L., Daly A.K., Day C.P., Anstee Q.M., Speliotes E.K. (2015). TM6SF2: catch-22 in the fight against nonalcoholic fatty liver disease and cardiovascular disease?. Gastroenterology.

[64] Lauridsen B.K., Stender S., Kristensen T., Fuglsang K.K., Kober L., Nordestgaard B. (2017). Liver fat content, NAFLD, and ischemic heart disease: Mendelian randomization and meta-analysis of 279,013 individuals. Eur Heart J.

[65] Speliotes E.K., Yerges-Armstrong L.M., Wu J., Hernaez R., Kim L.J., Palmer C.D. (2011). Genome-wide association analysis identifies variants associated with nonalcoholic fatty liver disease that have distinct effects on metabolic traits. PLoS Genet.

[66] Staley J.R., Blackshaw J., Kamat M.A., Ellis S., Surendran P., Sun B.B. (2016). PhenoScanner: a database of human genotype–phenotype associations. Bioinformatics.

[67] Yaghootkar H., Lotta L.A., Tyrrell J., Smit R.A.J., Jones S.E., Donnelly L. (2016). Genetic evidence for a link between favorable adiposity and lower risk of type 2 diabetes, hypertension, and heart disease. Diabetes.

[68] Newsome P.N., Cramb R., Davison S.M., Dillon J.F., Foulerton M., Godfrey E.M. (2018). Guidelines on the management of abnormal liver blood tests. Gut.

