# Corrigendum to: “Genome-wide and Mendelian randomisation studies of liver MRI yield insights into the pathogenesis of steatohepatitis” [J Hepatol (2020) 241-251]

**DOI:** 10.1016/j.jhep.2020.08.030

**Published:** 2020-12

**Authors:** Constantinos A. Parisinos, Henry R. Wilman, E. Louise Thomas, Matt Kelly, Rowan C. Nicholls, John McGonigle, Stefan Neubauer, Aroon D. Hingorani, Riyaz S. Patel, Harry Hemingway, Jimmy D. Bell, Rajarshi Banerjee, Hanieh Yaghootkar

**Affiliations:** 1 Institute of Health Informatics, Faculty of Population Health Sciences, University College London, London, UK; 2 Research Centre for Optimal Health, School of Life Sciences, University of Westminster, London, UK; 3 Perspectum Diagnostics Ltd., Oxford, UK; 4 Oxford Centre for Clinical Magnetic Resonance Research, Division of Cardiovascular Medicine, Oxford NIHR Biomedical Research Centre, University of Oxford, Oxford, UK; 5 Institute of Cardiovascular Science, Faculty of Population Health Sciences, University College London, London, UK; 6 Health Data Research UK London, Institute of Health Informatics, Faculty of Population Health Sciences, University College London, London, UK; 7 Genetics of Complex Traits, College of Medicine and Health, University of Exeter, Exeter, UK; 8 Division of Medical Sciences, Department of Health Sciences, Luleå University of Technology, Luleå, Sweden

It has come to our attention that there are errors in the Table 2 of the original manuscript “Genome-wide and Mendelian randomisation studies of liver MRI yield insights into the pathogenesis of steatohepatitis”.

**Table 2:** The association between 6 independent genetic variants and liver cT1. A linear mixed model was used for genetic associations (levels of significance: *p* <5 × 10^-8^).

SNP	CHR	Base pairs	EA	OA	EAF	Gene	Variant type	Amino acid change	BETA	Standard error	*p* value	Variance explained (%)
**rs759359281**	1	220,100,497	C	CA	0.06	*SLC30A10*	Intron		0.137	0.026	2.8 × 10^-8^	0.23
**rs13107325**	4	103,188,709	T	C	0.07	*SLC39A8*	Missense	A391T	0.544	0.022	1.2 × 10^-133^	3.95
**rs111723834**	14	24,572,932	A	G	0.02	*PCK2*	Missense, Intron	**R561Q**	0.291	0.046	3.0 × 10^-11^	0.27
**rs58542926**	19	19,379,549	T	C	0.07	*TM6SF2*	Missense	**E167K**	0.124	0.022	1.4 × 10^-8^	0.22
**rs4820268**∗	22	37,469,591	G	A	0.46	*TMPRSS6*	Synonymous	D521D	0.066	0.012	1.6 × 10^-9^	0.2
**rs738409**	22	44,324,727	G	C	0.21	*PNPLA3*	Missense	**I148M**	0.095	0.014	9.6 × 10^-13^	**0.29**

The coordinates and SNP IDs are in build 37. Effects are in SD. Beta represents effects in standard deviation (SD). CHR, chromosome; EA, effect allele; EAF, effect allele frequency; OA, other allele.

∗ rs4820268 is in LD (r^2^ = 0.77) with rs855791 (V736A).

The Variance Explained for SNP rs738409 has been incorrectly reported as 0.9. The correct value is 0.29. The amino acid changes for SNPs rs111723834, rs58542926 and rs738409 have been incorrectly reported as A561G, I148M and E167K, respectively. The correct amino acid changes are R561Q, E167K and I148M, respectively. SNP rs4820268 variant type (synonymous) and amino acid change (D521D) have also been corrected. Please see the corrected Table 2 below. These errors have occured during manual editing of the table and do not affect the results and conclusions of this article.

The authors would like to apologise for any inconvenience caused.

