# Supplementary material for: Genome-wide and Mendelian randomisation studies of liver MRI yield insights into the pathogenesis of steatohepatitis
Source: J Hepatol. 2020 Aug;73(2):241–51. doi: 10.1016/j.jhep.2020.03.032 (PMC7372222; doi:10.1016/j.jhep.2020.03.032)
Supplement: Supplementary information.pdf [file mmc1.pdf]

Genome-wide and Mendelian randomisation studies of liver MRI yield insights into the pathogenesis of steatohepatitis

Constantinos A Parisinos, Henry R Wilman, E Louise Thomas, Matt Kelly, Rowan C Nicholls, John McGonigle, Stefan Neubauer, Aroon D Hingorani, Riyaz S Patel, Harry Hemingway, Jimmy D Bell, Rajarshi Banerjee, Hanieh Yaghootkar

Table of contents

Supplementary Methods……………………………………………………………… 2

Fig. S1…………………………………………………………………………………...7

Fig. S2…………………………………………………………………………………...8

Fig. S3…………………………………………………………………………………...9

Fig. S4………………………………………………………………………………….10

Fig. S5………………………………………………………………………………….11

Table S1……………………………………………………………………………….12

Table S2……………………………………………………………………………….14

Table S3……………………………………………………………………………….15

Table S4……………………………………………………………………………….20

Table S5……………………………………………………………………………….20

Table S6……………………………………………………………………………….23

References…………………………………………………………………………….25

Supplementary Methods

The relationship between cT1 and Liver inflammation and Fibrosis (LIF) score.

A promising, non-invasive measure of steatohepatitis and fibrosis severity is magnetic resonance imaging (MRI) based corrected T1 (cT1).[1–3] T1 relaxation time reflects extracellular fluid which is characteristic of fibrosis and inflammation. The presence of iron, which can be determined from T2* maps, has an opposing effect. Combining T2* and T1 values can correct for this opposing effect, from which cT1 (in milliseconds) is derived. Higher cT1 values are associated with both histological liver inflammation and fibrosis, although their relative contributions to the score are still unknown.[3,4] cT1 has already been used as a non-invasive outcome in randomised controlled trials for non-alcoholic steatohepatitis (NASH)[5] and is associated with liver disease outcomes.[2]

cT1 is a continuous trait, and analysed as such in our GWAS in line with other continuous traits such as blood pressure, BMI and height.[6–8] In some earlier publications, cT1 was reported using the Liver Inflammation and Fibrosis (LIF) score. The LIF score is a tri-linear mapping of cT1 onto a continuous scale from 0 to 4 based on the association of cT1 with histological fibrosis.[3] LIF categories were defined as having no (LIF <1), mild (LIF 1–1.99), moderate (LIF 2–2.99), or severe (LIF 3–4) liver disease.[2] The LIF cut-off of 1.4 had a sensitivity of 91% and a specificity of 52% for the diagnosis of non-alcoholic steatohepatitis (NASH) versus steatosis (AUROC = 0.80), and corresponds to a cT1 value of 780ms; a slightly higher cutoff of 800ms is used in clinical trials[9] and is under evaluation by the FDA and European Medicines Agency as a diagnostic enrichment biomarker for NASH;[3,10] The LIF score is no longer used since the medical and MRI physics community is more familiar with T1 for the assessment of inflammation and fibrosis across all specialties including cardiology and neurology.[5,11–16] In this GWAS, the cT1 values reported

are standardised across the MRI scanner model and field strength and show very high repeatability and reproducibility.[17]

Principle of Mendelian Randomisation

Observational epidemiologic studies measure the association between an exposure and an outcome, however they are vulnerable to confounding, reverse causation and other forms of bias, and do not provide evidence that an observed association is causal. MR uses genetic variants associated with an exposure of interest (e.g. BMI, cholesterol, blood pressure) to assess its causal effect on an outcome of interest (e.g. steatohepatitis). In the classic MR paradigm, genetic associations are free from confounding since they are assigned randomly at conception from parents to offspring (according to Mendel's second law) and reverse causation is precluded since the sequence of the germline is not modifiable by disease. MR can be thought of as analogous to a randomised controlled trial (RCT) that uses naturally randomised genetic variation rather than randomised allocation to a drug or treatment, as the 'intervention'.[18]

Genetic polymorphisms that are associated with an exposure of interest are used as an instrument to randomly allocate study participants to higher or lower levels of the exposure under study. Because allocation to genetic variation in levels of the exposure is random, this study design should be less susceptible to confounding. In addition, the allocation of the polymorphism occurs at conception so this study design should not be vulnerable to reverse causation. The results of a Mendelian randomisation study can be interpreted as follows: If a polymorphism (or a collection of polymorphisms, a genetic instrument) is associated with an exposure and the outcome of interest, then the observed association between the exposure and outcome is likely to be causal. If not, then

the observed association between the exposure and outcome is likely to be an artefact of confounding, reverse causation or other study bias.

We used Mendelian randomisation to evaluate the causal association between multiple metabolic traits and diseases (e.g. insulin resistance, obesity, coronary artery disease) previously observationally associated with our outcome of interest (steatohepatitis). We were able to detect exposures that are likely to be causal (e.g. central obesity, insulin resistance, type 2 diabetes), protective (favourable adiposity), but also detect exposures where previous associations were likely to be down to confounding or other biases (e.g. blood pressure).

Mendelian Randomisation also has limitations and results need to be interpreted alongside other evidence in the field in the spirit of triangulation of evidence.[19] Despite presumed random allocation of genetic polymorphisms according to Mendel's law of independent assortment, this study design is still vulnerable to confounding e.g. by population structure or pleiotropy. Confounding by population structure can be addressed by performing studies within ethnically homogeneous study populations (as in this study). Confounding by pleiotropy can be addressed by selecting polymorphisms that are only associated with the exposure of interest, but not with other exposures that are known to be causally associated with the outcome under study, as well as using statistical methods (e.g. MR Egger) as this manuscript has done to correct for pleiotropy.

To create genetic instruments for possible causes of steatohepatitis, we constructed genetic scores for 24 predominantly metabolic traits. We combined multiple independently inherited polymorphisms to create genetic instruments. These genetic scores are instruments that reflect the combined effect of the polymorphisms included on the exposure of interest. As a result, each score has a much larger effect than any individual polymorphism included in the score. Genetic

instruments were constructed by using the effect sizes of independent, genome-wide significant genetic variants ($R^2 < 0.1$) associated with a particular exposure from previous GWASs. Using genetic instruments from a separate population compared to where the outcome of interest was measured results in less bias and more power, a method known as two-sample Mendelian randomisation.[20] We investigated the potential causal associations between 24 predominantly metabolic traits on cT1 using two-sample Mendelian randomisation analysis.

Supplementary Methods Table 1. List of genetic instruments used, number of SNPs comprising each genetic instrument, and PMIDs of the manuscripts from which the genetic instruments were derived.

Exposure	N_snp	PMID
Favourable adiposity	14	PMID: 30352878
Insulin resistance	53	PMID: 27841877
Insulin secretion	11	PMID: 22885924
Type 2 diabetes	89	PMID: 29632382
2 hour Glucose	8	PMID: 22885924
Fasting glucose	33	PMID: 22885924
Fasting Insulin	14	PMID: 22885924
NAFLD	4	PMID: 21423719
Body fat percentage	10	PMID: 26833246
BMI	73	PMID: 25673413
Coronary artery disease	64	PMID: 26343387
Systolic blood pressure	89	PMID: 27618452
Diastolic blood pressure	109	PMID: 27618452
LDL Cholesterol	80	PMID: 24097068

Triglycerides	62	PMID: 24097068
HDL Cholesterol	98	PMID: 24097068
Waist Hip ratio BMI adjusted	53	PMID: 30239722
Waist Hip ratio BMI adjusted (Female specific variants)	47	PMID: 30239722
Waist Hip ratio BMI adjusted (Male specific variants)	6	PMID: 30239722
Ferritin	6	PMID: 25352340
Transferrin	9	PMID: 25352340
Transferrin saturation	5	PMID: 25352340
Iron	5	PMID: 25352340
Height	809	PMID: 30124842

Fig. S1. Histograms of cT1 values in UK Biobank stratified by sex. 2.6% of women (169 / 6,455) and 5.3% of men (299 / 5,595) had values above 800ms, a threshold that has been set in current clinical trials as a cut-off for steatohepatitis (800ms shown by red dotted line).

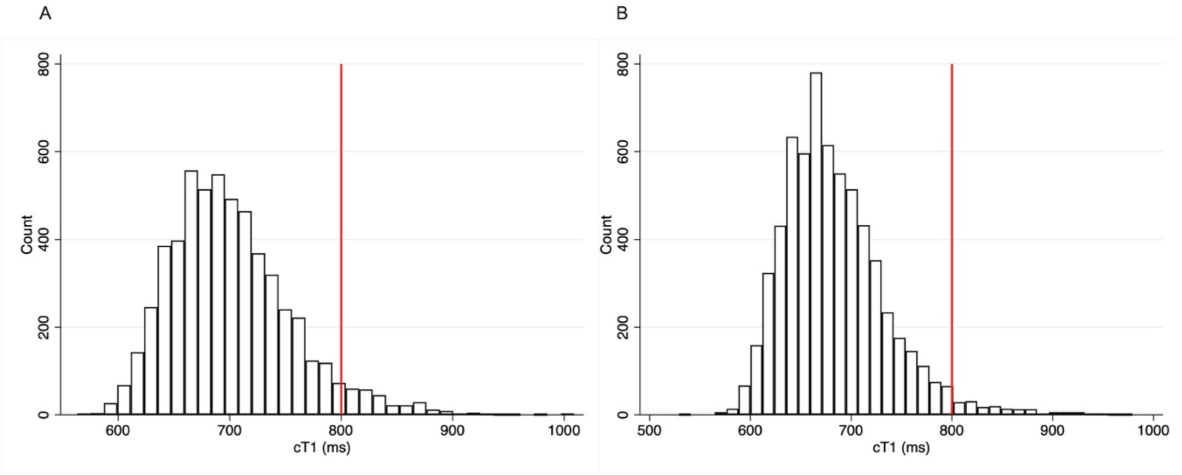

Fig. S2. Quantile quantile plot for cT1 GWAS. The observed versus expected -log10(p-values) in our GWAS supports normality and shows no evidence of inflation.

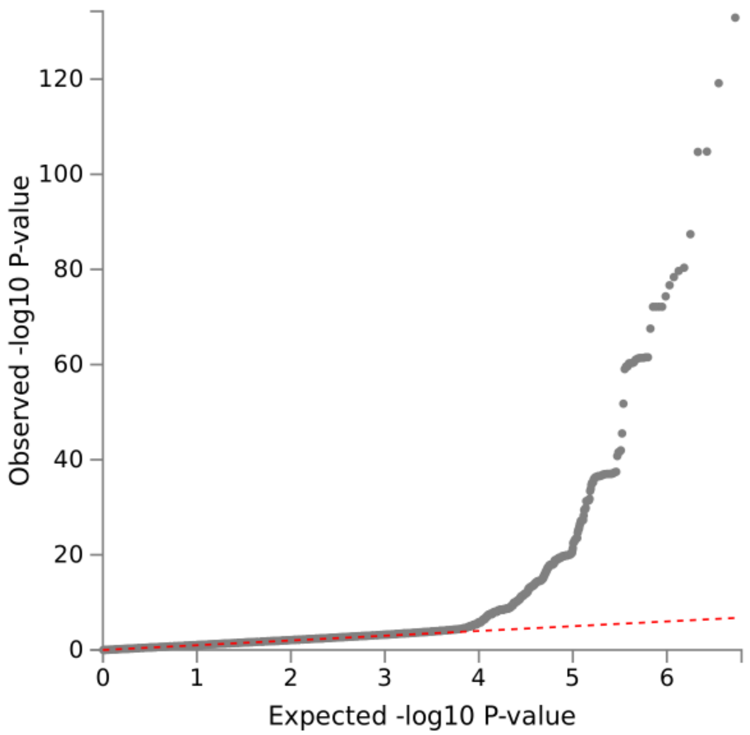

Fig. S3. cT1 values per *SLC39A8* genotype group. Red lines indicate the median values, blue lines indicate the lower and upper quartiles.

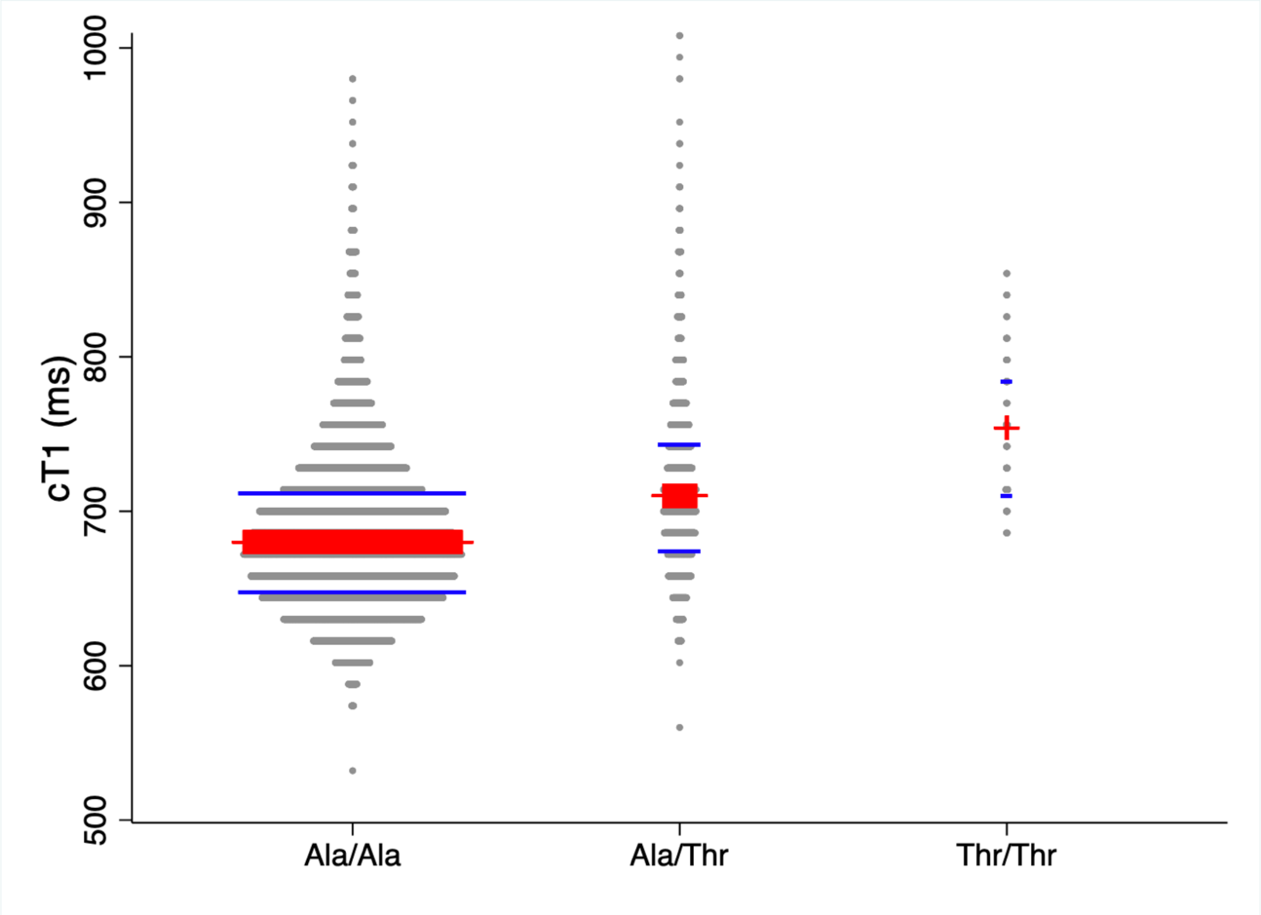

Fig. S4. Manhattan plot illustrating GWAS of liver PDFF measurements in 14,440 UK Biobank individuals (~12 million imputed variants). The x-axis is the chromosomal position and y-axis is the significance of association for each variant in log10(p-values). Grey line indicates genome-wide significance level. For the GWAS, a linear mixed model was used. Level of significance: $p < 5 \times 10^{-8}$.

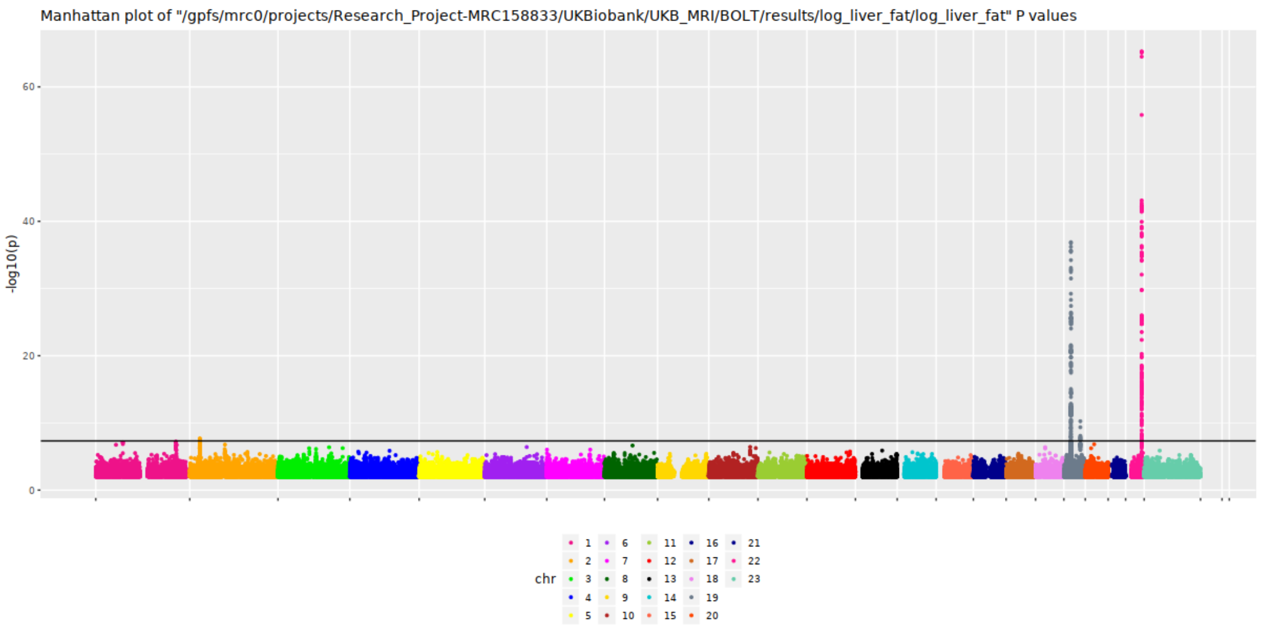

Fig. S5. Scatterplot of correlation between cT1 and a) BMI b) liver fat% (PDFF), and c) liver iron. P values calculated using t-test. Levels of significance: $p < 0.05$.

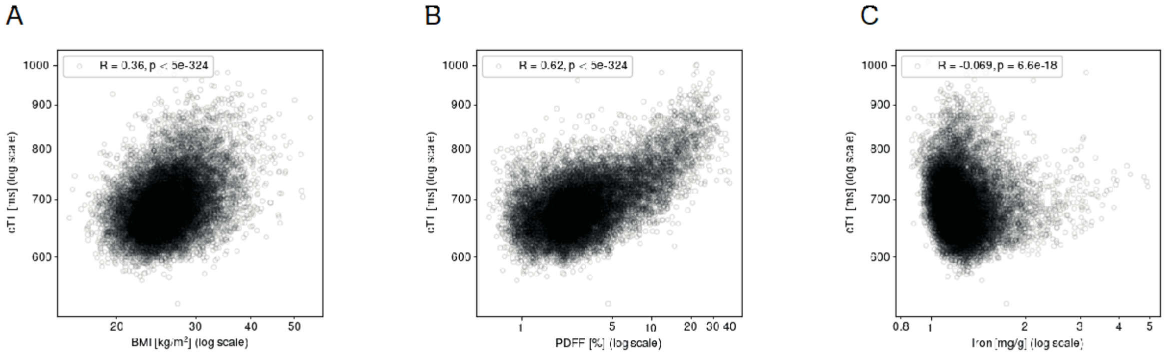

Table S1. GWAS Sensitivity analyses, with models correcting for a) BMI b) BMI & alcohol intake (in units) c) liver fat PDFF d) liver iron e) males only f) females only. A linear mixed model was used for genetic associations. Levels of significance: $p < 5 \times 10^{-8}$.

								cT1 (BMI) (N=14,405)		
**SNP**	**CHR**	**BP**	**EA**	**OA**	**EAF**	**Gene**	**variant type**	**BETA**	**SE**	**P**
**rs759359281**	1	220100497	C	CA	0.06	*SLC30A10*	Intron	0.131773	0.0242194	1.40E-08
**rs13107325**	4	103188709	T	C	0.07	*SLC39A8*	Missense Variant	0.530298	0.0210205	2.40E-143
**rs111723834**	14	24572932	A	G	0.02	*PCK2, NRL*	Missense Variant, Intron Variant	0.29092	0.0435268	2.20E-12
**rs58542926**	19	19379549	T	C	0.07	*TM6SF2*	Missense Variant	0.124756	0.0208328	1.10E-09
**rs4820268**	22	37469591	G	A	0.46	*TMPRSS6*	Missense Variant	0.108009	0.0132949	2.30E-17
**rs738409**	22	44324727	G	C	0.21	*PNPLA3*	Missense Variant	0.0656351	0.0109403	2.20E-10
								**cT1 (BMI & alcohol) (N=11,893)**		
								**BETA**	**SE**	**P**
**rs759359281**	1	220100497	C	CA	0.06	*SLC30A10*	Intron	0.13711	0.0266234	1.30E-07
**rs13107325**	4	103188709	T	C	0.07	*SLC39A8*	Missense Variant	0.536308	0.0229941	4.20E-122
**rs111723834**	14	24572932	A	G	0.02	*PCK2, NRL*	Missense Variant, Intron Variant	0.279295	0.0478867	4.40E-10
**rs58542926**	19	19379549	T	C	0.07	*TM6SF2*	Missense Variant	0.132978	0.0228134	5.00E-09
**rs4820268**	22	37469591	G	A	0.46	*TMPRSS6*	Missense Variant	0.121534	0.0146066	3.50E-18
**rs738409**	22	44324727	G	C	0.21	*PNPLA3*	Missense Variant	0.0643384	0.0119858	3.20E-08
								**cT1 (liver fat) (N=14,440)**		
								**BETA**	**SE**	**P**
**rs759359281**	1	220100497	C	CA	0.06	*SLC30A10*	Intron	0.126816	0.021926	1.70E-09
**rs13107325**	4	103188709	T	C	0.07	*SLC39A8*	Missense Variant	0.540024	0.0190318	5.60E-179

**rs111723834**	14	24572932	A	G	0.02	*PCK2, NRL*	Missense Variant, Intron Variant	0.308174	0.0394095	2.40E-16
**rs58542926**	19	19379549	T	C	0.07	*TM6SF2*	Missense Variant	-0.024769	0.0189835	1.90E-01
**rs4820268**	22	37469591	G	A	0.46	*TMPRSS6*	Missense Variant	0.0607271	0.00991015	5.10E-11
**rs738409**	22	44324727	G	C	0.21	*PNPLA3*	Missense Variant	-0.0308937	0.0121655	1.90E-02
								**cT1 (liver iron) (N=14,440)**		
								**BETA**	**SE**	**P**
**rs759359281**	1	220100497	C	CA	0.06	*SLC30A10*	Intron	0.136471	0.0254803	2.80E-08
**rs13107325**	4	103188709	T	C	0.07	*SLC39A8*	Missense Variant	0.541497	0.0221264	1.10E-133
**rs111723834**	14	24572932	A	G	0.02	*PCK2, NRL*	Missense Variant, Intron Variant	0.289944	0.0457921	2.9E-11
**rs58542926**	19	19379549	T	C	0.07	*TM6SF2*	Missense Variant	0.127372	0.0219207	4.00E-09
**rs4820268**	22	37469591	G	A	0.46	*TMPRSS6*	Missense Variant	0.0542081	0.0115437	7.40E-07
**rs738409**	22	44324727	G	C	0.21	*PNPLA3*	Missense Variant	0.0559764	0.01399	1.10E-12
								**cT1 (males) (N=6,640)**		
								**BETA**	**SE**	**P**
**rs759359281**	1	220100497	C	CA	0.06	*SLC30A10*	Intron	0.156337	0.0391243	5.20E-05
**rs13107325**	4	103188709	T	C	0.07	*SLC39A8*	Missense Variant	0.56999	0.0331774	1.10E-66
**rs111723834**	14	24572932	A	G	0.02	*PCK2, NRL*	Missense Variant, Intron Variant	0.344737	0.0711227	4.50E-07
**rs58542926**	19	19379549	T	C	0.07	*TM6SF2*	Missense Variant	0.161972	0.0334273	4.80E-07
**rs4820268**	22	37469591	G	A	0.46	*TMPRSS6*	Missense Variant	0.0674141	0.017446	9.30E-05
**rs738409**	22	44324727	G	C	0.21	*PNPLA3*	Missense Variant	0.121098	0.0211575	7.60E-09
								**cT1 (females) (N=7,800)**		
								**BETA**	**SE**	**P**

**rs759359281**	1	220100497	C	CA	0.06	*SLC30A10*	Intron	0.124803	0.0351335	2.60E-04
**rs13107325**	4	103188709	T	C	0.07	*SLC39A8*	Missense Variant	0.551638	0.0311469	2.90E-70
**rs111723834**	14	24572932	A	G	0.02	*PCK2, NRL*	Missense Variant, Intron Variant	0.268713	0.0625125	8.90E-06
**rs58542926**	19	19379549	T	C	0.07	*TM6SF2*	Missense Variant	0.0943526	0.0303544	2.10E-03
**rs4820268**	22	37469591	G	A	0.46	*TMPRSS6*	Missense Variant	0.0701305	0.0160201	7.20E-06
**rs738409**	22	44324727	G	C	0.21	*PNPLA3*	Missense Variant	0.0770984	0.0195081	2.60E-05

Table S2. The association between four independent genetic variants and liver PDFF in 14,440 UK Biobank participants. A linear mixed model was used for genetic associations. Levels of significance: $p < 5 \times 10^{-8}$.

SNP	CHR	BP	EA	OA	EAF	BETA	SE	P	Gene	Consequence
rs1260326	2	27730940	T	C	0.39117	0.046	0.009	3.90E-08	*GCKR*	Missense Variant
rs58542926	19	19379549	T	C	0.926558	0.206	0.016	6.30E-37	*TM6SF2*	Missense Variant
rs429358	19	45411941	T	C	0.848199	0.073	0.011	5.60E-11	*APOE*	Missense Variant
rs738409	22	44324727	G	C	0.788816	0.173	0.010	5.40E-66	*PNPLA3*	Missense Variant

Table S3. Associations of cT1 variants with liver blood tests, liver fat PDFF, liver iron content, cardiometabolic traits and diseases. A linear mixed model was used for genetic associations. Levels of significance: $p < 0.05$.

SNP	Chr	Outcome	BETA	SE	P	N
**rs759359281**	1	liver fat %	0.0127587	0.0253171	6.00E-01	14440
**rs759359281**	1	liver iron content	-7.53E-03	3.42E-02	8.26E-01	8282
**rs759359281**	1	ALT	0.01971676	0.00492174	0.00006175	361940
**rs759359281**	1	AST	0.02097398	0.00501843	0.00002924	360731
**rs759359281**	1	GGT	0.01092781	0.00485173	0.02430062	361888
**rs759359281**	1	ALP	0.01569743	0.00512336	0.0021849	362087
**rs759359281**	1	BMI	0.00760543	0.00506501	0.13321097	378214
**rs759359281**	1	HDL	-0.0109753	0.00494659	0.0265036	331494
**rs759359281**	1	LDL	0.01033718	0.00514988	0.04472196	361392
**rs759359281**	1	Triglycerides	0.01511628	0.00507773	0.00291123	361785
**rs759359281**	1	SHBG	-0.00536504	0.00499736	0.28301357	328442
**rs759359281**	1	Systolic blood pressure	-0.00570476	0.00463114	0.21801416	378821
**rs759359281**	1	Diastolic blood pressure	-0.00812936	0.00493904	0.09977707	378197
**rs759359281**	1	Type 2 diabetes	0.98768539	0.0207639	0.55066752	379703
**rs759359281**	1	Heart disease	1.0155469	0.01793243	0.38962282	379706
**rs759359281**	1	Hypertension	0.98441287	0.0128509	0.2215286	379708
**rs13107325**	4	liver fat %	0.00785646	0.0220298	6.40E-01	14440
**rs13107325**	4	liver iron content	-8.15E-02	3.00E-02	6.57E-03	8282
**rs13107325**	4	ALT	0.02205415	0.0042059	1.58E-07	361940

**rs13107325**	4	AST	0.06215688	0.00428884	1.39E-47	360731
**rs13107325**	4	GGT	-0.0247514	0.0041454	2.36E-09	361888
**rs13107325**	4	ALP	0.00261341	0.00437779	0.55052791	362087
**rs13107325**	4	BMI	0.05491581	0.00432855	7.11E-37	378214
**rs13107325**	4	HDL	-0.08265373	0.00422418	3.32E-85	331494
**rs13107325**	4	LDL	-0.03573906	0.00440007	4.59E-16	361392
**rs13107325**	4	Triglycerides	0.03163324	0.00433898	3.10E-13	361785
**rs13107325**	4	SHBG	-0.00723772	0.00426942	0.09002952	328442
**rs13107325**	4	Systolic blood pressure	-0.03389149	0.00395976	1.14E-17	378821
**rs13107325**	4	Diastolic blood pressure	-0.04711799	0.00422344	6.74E-29	378197
**rs13107325**	4	Type 2 diabetes	1.0718579	0.01719493	0.00005444	379703
**rs13107325**	4	Heart disease	0.98825314	0.0153871	0.44252172	379706
**rs13107325**	4	Hypertension	0.96248733	0.01100757	0.00051379	379708
**rs118045231**	10	liver fat %	0.0818138	0.0352523	9.30E-03	14440
**rs118045231**	10	liver iron content	-5.24E-02	4.83E-02	2.78E-01	8282
**rs118045231**	10	ALT	0.00166892	0.00648689	0.79696626	361940
**rs118045231**	10	AST	0.00438794	0.00661627	0.50720039	360731
**rs118045231**	10	GGT	-0.00173244	0.00639243	0.78638002	361888
**rs118045231**	10	ALP	0.00236123	0.00675068	0.72650622	362087
**rs118045231**	10	BMI	0.0032314	0.00666841	0.62797191	378214
**rs118045231**	10	HDL	-0.01015797	0.00652252	0.11938393	331494
**rs118045231**	10	LDL	-0.00854632	0.00678571	0.20786529	361392
**rs118045231**	10	Triglycerides	0.01254281	0.00669167	0.06087712	361785
**rs118045231**	10	SHBG	-0.00827293	0.00659444	0.20965037	328442

**rs118045231**	10	Systolic blood pressure	0.00405816	0.00609824	0.50575431	378821
**rs118045231**	10	Diastolic blood pressure	0.00047661	0.00650397	0.94158377	378197
**rs118045231**	10	Type 2 diabetes	1.0283803	0.02690784	0.29832486	379703
**rs118045231**	10	Heart disease	0.97960143	0.02383456	0.38720926	379706
**rs118045231**	10	Hypertension	1.0222345	0.01679198	0.1903289	379708
**rs111723834**	14	liver fat %	-0.0240158	0.0455128	6.70E-01	14440
**rs111723834**	14	liver iron content	-2.40E-02	6.18E-02	6.98E-01	8282
**rs111723834**	14	ALT	-0.01873864	0.00869896	0.03123117	361940
**rs111723834**	14	AST	-0.01608606	0.00887277	0.06983735	360731
**rs111723834**	14	GGT	-0.02653084	0.00857517	0.0019755	361888
**rs111723834**	14	ALP	-0.02270907	0.00905475	0.01214307	362087
**rs111723834**	14	BMI	-0.01252042	0.0089537	0.16200836	378214
**rs111723834**	14	HDL	0.03186024	0.0087415	0.00026773	331494
**rs111723834**	14	LDL	-0.00258033	0.00910383	0.77684502	361392
**rs111723834**	14	Triglycerides	-0.03984006	0.00897427	9.03E-06	361785
**rs111723834**	14	SHBG	0.03205497	0.00882511	0.00028101	328442
**rs111723834**	14	Systolic blood pressure	-0.01311047	0.00818987	0.10941868	378821
**rs111723834**	14	Diastolic blood pressure	-0.02248176	0.008737	0.01007761	378197
**rs111723834**	14	Type 2 diabetes	0.91055144	0.0378863	0.01338647	379703
**rs111723834**	14	Heart disease	0.97187684	0.03207806	0.37385583	379706
**rs111723834**	14	Hypertension	0.92386097	0.0230377	0.00058696	379708
**rs58542926**	19	liver fat %	0.261101	0.0217732	8.10E-32	14440
**rs58542926**	19	liver iron content	5.22E-02	2.93E-02	7.48E-02	8282
**rs58542926**	19	ALT	0.08092395	0.00418902	4.17E-83	361940

**rs58542926**	19	AST	0.05932545	0.00427257	8.00E-44	360731
**rs58542926**	19	GGT	0.01636427	0.00413194	0.00007483	361888
**rs58542926**	19	ALP	-0.09005039	0.00436066	1.09E-94	362087
**rs58542926**	19	BMI	-0.00326425	0.00431546	0.4494056	378214
**rs58542926**	19	HDL	-0.00408918	0.00420784	0.33115097	331494
**rs58542926**	19	LDL	-0.13974327	0.00437875	3.51E-223	361392
**rs58542926**	19	Triglycerides	-0.10360269	0.00432086	6.02E-127	361785
**rs58542926**	19	SHBG	-0.00340488	0.00425019	0.42306731	328442
**rs58542926**	19	Systolic blood pressure	0.01470579	0.00394581	0.00019385	378821
**rs58542926**	19	Diastolic blood pressure	0.02296663	0.00420855	4.84E-08	378197
**rs58542926**	19	Type 2 diabetes	1.0846765	0.01710389	2.01E-06	379703
**rs58542926**	19	Heart disease	0.95968678	0.01551553	0.00799994	379706
**rs58542926**	19	Hypertension	1.024177	0.01084989	0.02767896	379708
**rs4820268**	22	liver fat %	0.0123012	0.0114339	3.20E-01	14440
**rs4820268**	22	liver iron content	-1.04E-01	1.55E-02	2.63E-11	8282
**rs4820268**	22	ALT	-0.0039429	0.00221457	0.07500625	361940
**rs4820268**	22	AST	-0.00979042	0.00225823	0.00001455	360731
**rs4820268**	22	GGT	-0.0024286	0.002183	0.26592274	361888
**rs4820268**	22	ALP	-0.00683427	0.00230523	0.00303023	362087
**rs4820268**	22	BMI	-0.00240988	0.00227925	0.29036959	378214
**rs4820268**	22	HDL	-0.00047719	0.00222443	0.83014014	331494
**rs4820268**	22	LDL	0.00764322	0.00231692	0.00097086	361392
**rs4820268**	22	Triglycerides	-0.00949943	0.00228489	0.00003218	361785
**rs4820268**	22	SHBG	-0.00500005	0.00224719	0.0260806	328442

**rs4820268**	22	Systolic blood pressure	0.00294821	0.00208454	0.15726715	378821
**rs4820268**	22	Diastolic blood pressure	-0.00187424	0.00222338	0.39924638	378197
**rs4820268**	22	Type 2 diabetes	1.000291	0.00930691	0.97502587	379703
**rs4820268**	22	Heart disease	1.019562	0.00810697	0.01686224	379706
**rs4820268**	22	Hypertension	1.012714	0.0057664	0.02845281	379708
**rs738409**	22	liver fat %	0.233979	0.0138955	1.20E-63	14440
**rs738409**	22	liver iron content	5.36E-02	1.89E-02	4.66E-03	8282
**rs738409**	22	ALT	0.09831972	0.00267411	2.18E-295	361940
**rs738409**	22	AST	0.09945885	0.00272673	9.25E-291	360731
**rs738409**	22	GGT	-0.00130433	0.00264091	0.62138227	361888
**rs738409**	22	ALP	-0.01233248	0.00278871	9.77E-06	362087
**rs738409**	22	BMI	-0.00899027	0.00275861	0.00111822	378214
**rs738409**	22	HDL	-0.01596964	0.00269179	2.98E-09	331494
**rs738409**	22	LDL	-0.01270562	0.00280268	5.81E-06	361392
**rs738409**	22	Triglycerides	-0.00292861	0.00276422	0.28938526	361785
**rs738409**	22	SHBG	0.04467104	0.00271863	1.20E-60	328442
**rs738409**	22	Systolic blood pressure	-0.00002693	0.00252259	0.99148313	378821
**rs738409**	22	Diastolic blood pressure	0.00254793	0.00269094	0.34371227	378197
**rs738409**	22	Type 2 diabetes	1.0346154	0.01116244	0.00229915	379703
**rs738409**	22	Heart disease	0.97555595	0.00985126	0.01200007	379706
**rs738409**	22	Hypertension	0.99394356	0.00697888	0.38404798	379708

Table S4. The association between cT1 variants and ALT / AST measures in Chambers *et al*.[21]

SNP	CHR	EA	OA	EAF	Gene	Beta ALT	SE ALT	P ALT	Beta AST	SE AST	P AST
**rs759359281**	1	C	CA	0.06	*SLC30A10*	NA	NA	NA	NA	NA	NA
**rs13107325**	4	T	C	0.07	*SLC39A8*	0.01	0.005	0.2745	0.0139	0.004	0.005
**rs111723834**	14	A	G	0.02	*PCK2, NRL*	NA	NA	NA	NA	NA	NA
**rs10401969 (rs58542926 proxy)**	19	C	T	0.88	*TM6SF2*	0.0111	0.0038	0.0003	0.0164	0.004	0.00004
**rs4820268**	22	G	A	0.46	*TMPRSS6*	0.0001	0.0016	0.515	0.0012	0.002	0.189
**rs738409**	22	G	C	0.21	*PNPLA3*	0.0254	0.0021	1.21E-45	0.0254	0.0023	1.23E-34

Table S5. Genetic correlation analyses between cT1 measures and 120 predominantly metabolic traits. For clarity, we present traits where $p < 0.05$ (t-test, levels of significance: $p < 0.01$).

Phenotype	PMID	rg	SE	P
HOMA-IR	20081858	0.5272	0.1489	0.0004
Mean diameter for VLDL particles	27005778	0.5207	0.146	0.0004
Glycoprotein acetyls; mainly a1-acid glycoprotein	27005778	0.5027	0.1566	0.0013
Triglycerides in small HDL	27005778	0.4957	0.1897	0.009
Leucine	27005778	0.4918	0.2024	0.0151
Tyrosine	27005778	0.4548	0.1755	0.0096
Isoleucine	27005778	0.4422	0.1724	0.0103
Fasting insulin main effect	22581228	0.4277	0.1158	0.0002

Phenylalanine	27005778	0.4266	0.2047	0.0371
Triglycerides in medium VLDL	27005778	0.4247	0.1547	0.0061
Type 2 Diabetes	22885922	0.4239	0.1043	4.79E-05
Triglycerides in small VLDL	27005778	0.4116	0.1423	0.0038
Concentration of medium VLDL particles	27005778	0.4041	0.1348	0.0027
Valine	27005778	0.4034	0.1829	0.0274
Waist circumference	25673412	0.3917	0.0699	2.09E-08
Body fat	26833246	0.388	0.0825	2.59E-06
Total lipids in medium VLDL	27005778	0.3876	0.138	0.005
Body mass index	20935630	0.3856	0.061	2.57E-10
HOMA-B	20081858	0.3842	0.1181	0.0011
Triglycerides in large VLDL	27005778	0.3841	0.1419	0.0068
Total lipids in large VLDL	27005778	0.3802	0.1393	0.0063
Concentration of very large VLDL particles	27005778	0.3752	0.1435	0.0089
Phospholipids in chylomicrons and largest VLDL particles	27005778	0.3727	0.1499	0.0129
Waist-to-hip ratio	25673412	0.3658	0.0749	1.05E-06
Total cholesterol in large VLDL	27005778	0.3603	0.1341	0.0072
Total lipids in very large VLDL	27005778	0.3552	0.1381	0.0101
Concentration of large VLDL particles	27005778	0.3501	0.1472	0.0174
Triglycerides in chylomicrons and largest VLDL particles	27005778	0.3464	0.1544	0.0249
Concentration of small VLDL particles	27005778	0.3443	0.1317	0.009
Serum total triglycerides	27005778	0.3422	0.1385	0.0135
Total lipids in chylomicrons and largest VLDL particles	27005778	0.3325	0.15	0.0266
Obesity class 2	23563607	0.3294	0.0746	1.01E-05
Phospholipids in large VLDL	27005778	0.3276	0.1399	0.0192
Free cholesterol in large VLDL	27005778	0.3249	0.1315	0.0135
Triglycerides in very large VLDL	27005778	0.3247	0.1299	0.0124
Phospholipids in medium VLDL	27005778	0.3244	0.1429	0.0232
Phospholipids in very large VLDL	27005778	0.3237	0.1428	0.0234
Total lipids in small VLDL	27005778	0.3235	0.1331	0.0151

Cholesterol esters in large VLDL	27005778	0.3149	0.1277	0.0137
Concentration of chylomicrons and largest VLDL particles	27005778	0.3128	0.1403	0.0257
Average number of methylene groups per a double bond	27005778	0.303	0.1268	0.0169
Phospholipids in small VLDL	27005778	0.2978	0.1374	0.0302
Free cholesterol in medium VLDL	27005778	0.2928	0.1409	0.0377
Total cholesterol in medium VLDL	27005778	0.2909	0.1358	0.0322
Hip circumference	25673412	0.2898	0.0616	2.56E-06
Obesity class 3	23563607	0.2765	0.1105	0.0123
HbA1C	20858683	0.275	0.1233	0.0258
Cholesterol esters in medium VLDL	27005778	0.2624	0.1325	0.0476
Crohns disease	26192919	0.1897	0.0854	0.0263
Coronary artery disease	26343387	0.1822	0.0669	0.0065
Triglycerides	20686565	0.1508	0.0762	0.0476
Ratio of bisallylic groups to double bonds	27005778	-0.2844	0.1073	0.008
Ratio of bisallylic groups to total fatty acids	27005778	-0.2893	0.117	0.0134
HDL cholesterol	20686565	-0.307	0.0686	7.58E-06
Average number of double bonds in a fatty acid chain	27005778	-0.3073	0.1307	0.0187
Citrate	27005778	-0.3293	0.1562	0.0351
Apolipoprotein A-I	27005778	-0.4093	0.1882	0.0296
Acetate	27005778	-0.4596	0.1846	0.0128
Total cholesterol in HDL	27005778	-0.4876	0.1594	0.0022
Total lipids in large HDL	27005778	-0.5206	0.1392	0.0002
Concentration of very large HDL particles	27005778	-0.5545	0.215	0.0099
Phospholipids in very large HDL	27005778	-0.5554	0.1692	0.001
Total lipids in very large HDL	27005778	-0.6045	0.2178	0.0055
Free cholesterol in very large HDL	27005778	-0.6146	0.2366	0.0094
Total cholesterol in very large HDL	27005778	-0.6232	0.3029	0.0396

Table S6. Mendelian randomisation sensitivity analyses. Egger test, weighted median (WM) and penalised weighted median (PWM) show similar directional effects with the IVW method. Levels of significance: $p < 0.05$.

Exposure	betaEgger	pEgger	betaWM	pWM	betaPWM	pPWM	N_snp	PMID
Favourable adiposity	-0.675	0.201	-0.328	0.149	-0.284	0.221	14	PMID: 30352878
Insulin resistance	0.327	0.337	0.512	0.001	0.488	0.001	53	PMID: 27841877
Insulin secretion	0.117	0.697	0.067	0.729	0.123	0.501	11	PMID: 22885924
Type 2 diabetes	0.072	0.1312	0.055	0.068	0.053	0.089	89	PMID: 29632382
2 hour Glucose	-0.055	0.903	0.015	0.851	-0.007	0.931	8	PMID: 22885924
Fasting glucose	0.0382	0.855	-0.176	0.221	-0.172	0.258	33	PMID: 22885924
Fasting Insulin	-4.555	0.014	0.792	0.014	0.838	0.008	14	PMID: 22885924
NAFLD	0.391	0.107	0.365	4.06E-11	-0.049	0.806	4	PMID: 21423719
Body fat percentage	1.173	0.160	0.430	0.008	0.435	0.007	10	PMID: 26833246
BMI	0.355	0.014	0.251	0.007	0.242	0.005	73	PMID: 25673413
Coronary artery disease	-0.025	0.635	-0.036	0.266	-0.045	0.202	64	PMID: 26343387
Systolic blood pressure	-0.046	0.090	-0.002	0.554	-0.011	0.055	89	PMID: 27618452
Diastolic blood pressure	-0.099	0.006	-0.012	0.087	-0.013	0.117	109	PMID: 27618452
LDL Cholesterol	-0.031	0.675	-0.035	0.504	-0.035	0.503	80	PMID: 24097068
Triglycerides	-0.127	0.170	-0.079	0.219	-0.067	0.424	62	PMID: 24097068
HDL Cholesterol	0.033	0.796	0.022	0.646	0.022	0.646	98	PMID: 24097068
Waist Hip ratio BMI adjusted	0.100	0.701	0.072	0.471	0.015	0.881	53	PMID: 30239722
Waist Hip ratio BMI adjusted (Female specific variants)	0.087	0.537	0.247	0.001	0.181	0.012	47	PMID: 30239722
Waist Hip ratio BMI adjusted (Male specific variants)	3.905	0.269	0.187	0.435	0.017	0.947	6	PMID: 30239722
Ferritin	-0.372	0.329	-0.567	8.35E-08	-0.100	0.521	6	PMID: 25352340
Transferrin	0.039	0.679	0.092	0.032	0.025	0.443	9	PMID: 25352340

Transferrin saturation	-0.228	0.029	-0.238	1.48E-11	-0.100	0.378	5	PMID: 25352340
Iron	-0.464	0.009	-0.332	1.72E-12	0.0117	0.942	5	PMID: 25352340
Height	-0.087	0.150	0.001	0.966	0.0124	0.6476	809	PMID: 30124842

References

[1] Pavlides M, Banerjee R, Tunnicliffe EM, Kelly C, Collier J, Wang LM, et al. Multiparametric magnetic resonance imaging for the assessment of non-alcoholic fatty liver disease severity. Liver Int. 2017;37:1065–1073.

[2] Pavlides M, Banerjee R, Sellwood J, Kelly CJ, Robson MD, Booth JC, et al. Multiparametric magnetic resonance imaging predicts clinical outcomes in patients with chronic liver disease. J. Hepatol. 2016;64:308–315.

[3] Banerjee R, Pavlides M, Tunnicliffe EM, Piechnik SK, Sarania N, Philips R, et al. Multiparametric magnetic resonance for the non-invasive diagnosis of liver disease. J. Hepatol. 2014;60:69–77.

[4] Cheung A, Neuschwander-Tetri BA, Kleiner DE, Schabel E, Rinella M, Harrison S, et al. Defining Improvement in Nonalcoholic Steatohepatitis for Treatment Trial Endpoints: Recommendations from the Liver Forum. Hepatology [Internet]. 2019;Available from: http://dx.doi.org/10.1002/hep.30672

[5] Harrison SA, Rossi SJ, Paredes AH, Trotter JF, Bashir MR, Guy CD, et al. NGM282 Improves Liver Fibrosis and Histology in 12 Weeks in Patients with Nonalcoholic Steatohepatitis. Hepatology [Internet]. 2019;Available from: http://dx.doi.org/10.1002/hep.30590

[6] Giri A, Hellwege JN, Keaton JM, Park J, Qiu C, Warren HR, et al. Trans-ethnic association study of blood pressure determinants in over 750,000 individuals. Nat. Genet. 2019;51:51–62.

[7] Yengo L, Sidorenko J, Kemper KE, Zheng Z, Wood AR, Weedon MN, et al. Meta-analysis of genome-wide association studies for height and body mass index in ∼700000 individuals of European ancestry. Hum. Mol. Genet. 2018;27:3641–3649.

[8] Cantor RM, Lange K, Sinsheimer JS. Prioritizing GWAS results: A review of statistical methods and recommendations for their application. Am. J. Hum. Genet. 2010;86:6–22.

[9] Non-invasive Rapid Assessment of NAFLD Using Magnetic Resonance Imaging With LiverMultiScan - Full Text View - ClinicalTrials.gov [Internet]. [cited 2019 Jun 11];Available from: https://clinicaltrials.gov/ct2/show/NCT03289897

[10] Pavlides M, Banerjee R, Kelly CJ, Robson MD, Neubauer S, Barnes E. Reply to: “Multiparametric magnetic resonance imaging to predict clinical outcomes in patients with chronic liver disease: A cautionary note on a promising technique.” J. Hepatol. 2017;66:457–458.

[11] Harrison SA, Dennis A, Fiore MM, Kelly MD, Kelly CJ, Paredes AH, et al. Utility and variability of three non-invasive liver fibrosis imaging modalities to evaluate efficacy of GR-MD-02 in subjects with NASH and bridging fibrosis during a phase-2 randomized clinical trial. PLoS One. 2018;13:e0203054.

[12] Mojtahed A, Kelly CJ, Herlihy AH, Kin S, Wilman HR, McKay A, et al. Reference range of liver corrected T1 values in a population at low risk for fatty liver disease-a UK Biobank sub-study, with an appendix of interesting cases. Abdom Radiol (NY). 2019;44:72–84.

[13] McDonald N, Eddowes PJ, Hodson J, Semple SIK, Davies NP, Kelly CJ, et al. Multiparametric magnetic resonance imaging for quantitation of liver disease: a two-centre cross-sectional observational study. Sci. Rep. 2018;8:9189.

[14] Ostovaneh MR, Ambale-Venkatesh B, Fuji T, Bakhshi H, Shah R, Murthy VL, et al. Association of Liver Fibrosis With Cardiovascular Diseases in the General Population: The Multi-Ethnic Study of Atherosclerosis (MESA). Circ. Cardiovasc. Imaging. 2018;11:e007241.

[15] Liu C-Y, Liu Y-C, Wu C, Armstrong A, Volpe GJ, van der Geest RJ, et al. Evaluation of age-related interstitial myocardial fibrosis with cardiac magnetic resonance contrast-enhanced T1 mapping: MESA (Multi-Ethnic Study of Atherosclerosis). J. Am. Coll. Cardiol. 2013;62:1280–1287.

[16] Mealer RG, Jenkins BG, Chen C-Y, Daly MJ, Ge T, Lehoux S, et al. A schizophrenia risk locus alters brain metal transport and plasma glycosylation [Internet]. bioRxiv. 2019 [cited 2019 Sep 26];757088. Available from: https://www.biorxiv.org/content/10.1101/757088v1

[17] Bachtiar V, Kelly MD, Wilman HR, Jacobs J, Newbould R, Kelly CJ, et al. Repeatability and reproducibility of multiparametric magnetic resonance imaging of the liver. PLoS One. 2019;14:e0214921.

[18] Hingorani A, Humphries S. Nature's randomised trials. Lancet. 2005;366:1906–1908.

[19] Lawlor DA, Tilling K, Davey Smith G. Triangulation in aetiological epidemiology. Int. J. Epidemiol. 2016;45:1866–1886.

[20] Pierce BL, Burgess S. Efficient Design for Mendelian Randomization Studies: Subsample and 2-Sample Instrumental Variable Estimators. Am. J. Epidemiol. 2013;178:1177–1184.

[21] Chambers JC, Zhang W, Sehmi J, Li X, Wass MN, Van der Harst P, et al. Genome-wide association study identifies loci influencing concentrations of liver enzymes in plasma. Nat. Genet. 2011;43:1131–1138.
